# Cardiovascular Profile of Xanthone-Based 1,4 Dihydropyridines Bearing a Lidoflazine Pharmacophore Fragment

**DOI:** 10.3390/molecules23123088

**Published:** 2018-11-27

**Authors:** Alessandra Bisi, Matteo Micucci, Silvia Gobbi, Federica Belluti, Roberta Budriesi, Angela Rampa

**Affiliations:** Department of Pharmacy and Biotechnology, Alma Mater Studiorum University of Bologna, Via Belmeloro, 6, 40126 Bologna, Italy; matteo.micucci2@unibo.it (M.M.); silvia.gobbi@unibo.it (S.G.); federica.belluti@unibo.it (F.B.); roberta.budriesi@unibo.it (R.B.); angela.rampa@unibo.it (A.R.)

**Keywords:** xanthone, 1,4-dihydropyridine, lidoflazine, calcium channels, hybrid compounds

## Abstract

As a follow-up to our previous studies on differently substituted 1,4-dihydropyridines endowed with a peculiar cardiac selectivity, in this paper, a small series of hybrid compounds bearing the pharmacophore fragment of lidoflazine in position 2 or 3 on a 4-(xanthen-9-one)-dihydropyridine core was reported. Lidoflazine was selected due to our promising previously reported data, and the xanthen-9-one substituent was introduced in position 4 of the dihydropyridine scaffold based on the cardiac selectivity observed in several of our studies. The new hybrid compounds were tested to assess cardiac and vascular activities, and the data were evaluated in comparison with those previously obtained for 4-(xanthen-9-one)-dihydropyridines and lidoflazine–nifedipine hybrid compounds. The functional studies indicated an interesting peculiar selectivity for the cardiac parameter inotropy, in particular when the lidoflazine fragment was introduced in position 2 of the dihydropyridine scaffold (**4a**–**e**), and thus a possible preferential binding with the Ca_v_ 1.2 isoform of l-type calcium channels, which are mainly involved in cardiac contractility.

## 1. Introduction

Arrhythmia and hypertension are considered the most relevant factors contributing to cardiovascular diseases (CVDs), which currently still represent the main cause of worldwide mortality [1]. Arrhythmia has been defined as a defect in the sequence of electrical impulses occurring in the course of the cardiac cycle, while hypertension, or abnormally high blood pressure, can be deemed the most common chronic condition, affecting billions of people around the world [2].

Undoubtedly, ion channels, mainly sodium, potassium, and calcium channels, play a pivotal role in the regulation of electrical activity in cardiac cells, and aberrations in their functions or expressions account for the onset of cardiac arrhythmia [3]. The role of calcium channels (CCs) in hypertension is also widely recognized, and the ‘2013 Guidelines for the Management of Arterial Hypertension’ [4], published by the European Society of Hypertension (ESH) together with the European Society of Cardiology (ESC), recommended calcium channel blockers (CCBs) as the initial therapy, alone or in combination with other drug classes. Indeed, despite the increasing number of marketed drugs able to target the different mechanisms involved in this CVD, efficient pressure regulation often requires a combination of drugs.

The CCs mainly involved in cardiac and vascular regulation are the so-called long-lasting, “l-type” CCs (LTCCs), which are voltage-sensitive complex proteins formed by different distinct subunits. The Ca^2+^-selective pores of all voltage-gated Ca^2+^ channels are comprised by α1 subunits, of which four isoforms (Ca_v_ 1.1–Ca_v_ 1.4) have been reported, according to their differential tissue distribution. While Ca_v_ 1.1 is mainly expressed in skeletal muscle and Ca_v_ 1.4 is predominantly found in the retina, Ca_v_ 1.2 and Ca_v_ 1.3 are located in most electrically excitable cells, often both in the same cell, in tissues such as the brain and heart, making these LTCC isoforms attractive drug targets [5,6].

**Figure 1:**
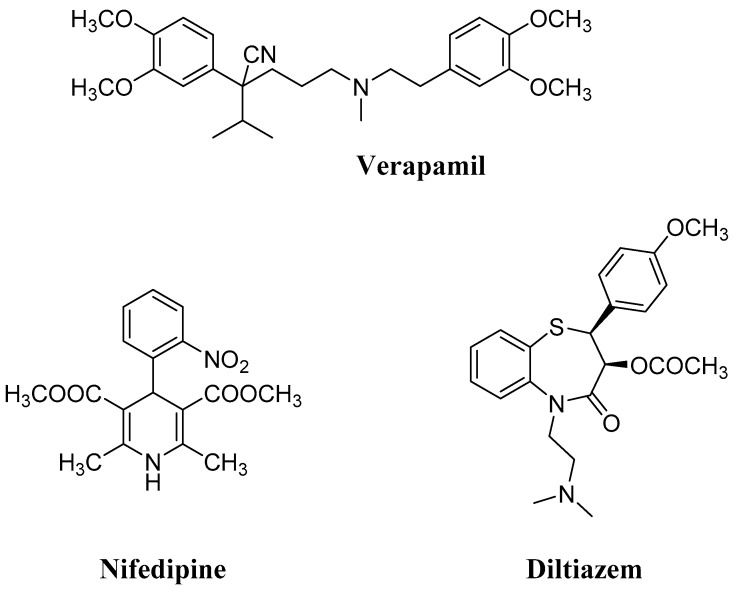
Prototypes of the main classes of calcium channel blockers (CCBs).

CCBs can be classified into three main unrelated chemical classes (Figure 1), namely phenylalkylamines (prototype verapamil), benzothiazepines (prototype diltiazem), and 1,4-dihydropyridines (1,4-DHPs, prototype nifedipine), all binding within a single overlapping drug-binding region located in the pore-forming α1 subunit of the LTCC. Through this interaction, CCBs interfere with the voltage-dependent cycling of the channel (in particular, 1,4-DHPs showed higher affinity for the inactivated state of the channel, prevailing in arterial smooth muscle [7]), leading to a “voltage-dependent” block. Because of their prevalent binding with the Ca_v_ 1.2 isoform, 1,4-DHPs induce arterial vasodilation, and by decreasing peripheral vascular resistance and afterload, they also reduce cardiac oxygen demand. Usually, differently from verapamil and diltiazem, they lack appreciable negative inotropic actions at therapeutic doses [6]. Due to this peculiar selectivity, 1,4-DHPs represent the most prescribed CCBs in hypertension, and to date, second-, third-, and fourth-generation 1,4-DHPs have been developed, starting from the prototype nifedipine. Over the years, the structure of this prominent drug has been deeply investigated and extensively modified in order to improve its potency and pharmacokinetic profile. In particular, the introduction of large lipophilic esters in positions 3 or 5, as well as the modification of the substituted aromatic group in position 4, were among the most successful structural modifications, leading to new potent marketed CCBs [8].

In the last decade, the concept of polypharmacology, namely the ability of a single drug to engage different targets involved in complex diseases, has gained ever-increasing interest, mainly as a promising strategy to improve efficacy [9]. In this framework, hybrid molecules obtained by the combination of properly selected pharmacophores of two differently active compounds could represent a valuable approach to develop new molecular entities with improved biological properties [10].

**Figure 2:**
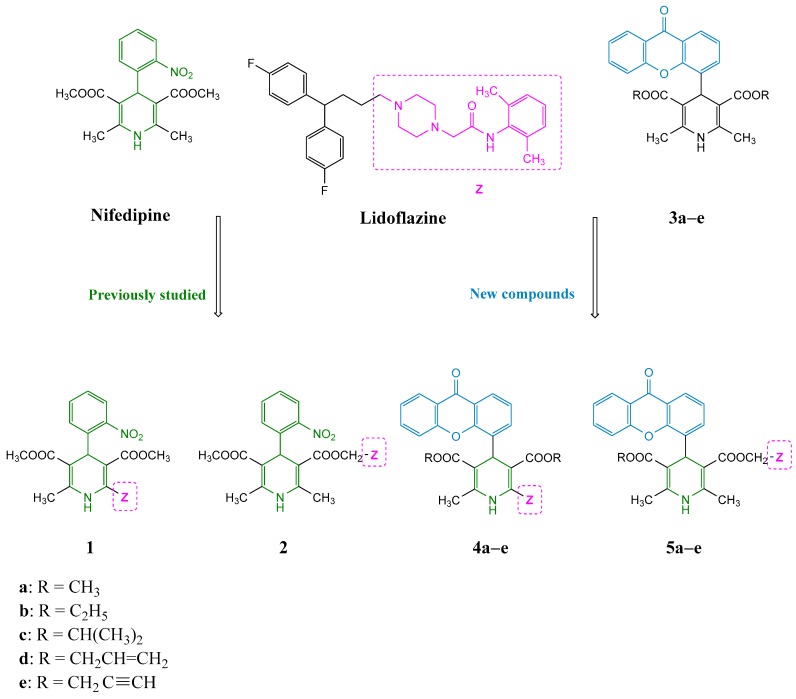
Design strategy for the studied compounds **4a**–**e** and **5a**–**e**.

Some years ago, we reported a series of 1,4-DHPs structurally related to nifedipine and bearing a pharmacophore fragment of lidoflazine, an atypical calcium channel modulator, in different positions of the DHP scaffold (**1** and **2**, see Figure 2) [11]. These hybrid compounds showed increased negative inotropic and chronotropic effects with respect to nifedipine itself, in particular when the lidoflazine fragment was inserted in position 3 of the DHP nucleus. Moreover, this modification led to a marked decrease in the vascular smooth muscle calcium antagonistic effect, indicating an interesting cardiac selectivity. In our long-lasting experience in the design and synthesis of DHPs, we have also reported Structure-Activity Relationship (SAR) studies on different series of 4-(xanthen-9-one)-DHP derivatives endowed with an unusual selectivity for cardiac functional parameters, namely chronotropy and inotropy, with respect to vascular ones (xanthodipine **3a** and congeners, see Figure 2) [12,13,14,15].

In this paper, based on previously reported results and aiming at extending our studies on the cardiac selectivity of these derivatives, a small series of hybrid compounds was designed, in which the peculiar portion of lidoflazine was introduced in position 2 or 3 on the 4-(xanthen-9-one)-DHP core (Figure 2). Lidoflazine was selected due to its multifaceted profile: this diphenylalkylpiperazine derivative, clinically used in some countries as an antianginal agent, is a long-acting coronary vasodilator and its antiarrhythmic properties in animals have also been described [16]. Additional intracellular mechanisms besides calcium entry blockade are supposed to be involved in its smooth muscle relaxant activity, in particular the inhibition of nucleoside transporters, which leads to an increase in adenosine production that contributes to the vasodilator effect [17].

The new hybrid compounds were tested in functional studies to evaluate their cardiac and vascular activities. To assess the role of the xanthen-9-one (Xant) substituent, the biological data of the previously described lidoflazine–nifedipine hybrid compounds **1** and **2** were also reported, as well as the data of xanthodipine and related compounds **3a**–**e**, bearing the same R substituents, to evaluate the effect of the introduction of the lidoflazine fragment. The chemical structures of all compounds tested in this study are reported in Figure 2.

## 2. Results

### 2.1. Chemistry

**Scheme 1:**
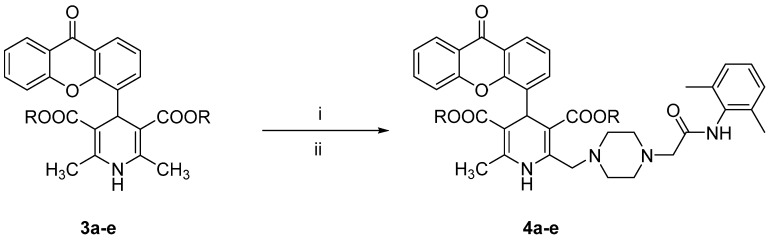
Reagents and conditions: (i) pyridinium bromide perbromide, CH_2_Cl_2_, pyridine, 0 °C, 40 min; (ii) *N*-(2,6-dimethyl-phenyl)-2-piperazin-1-ylacetamide, K_2_CO_3_, THF/DMF, room temperature, overnight.

**Scheme 2:**
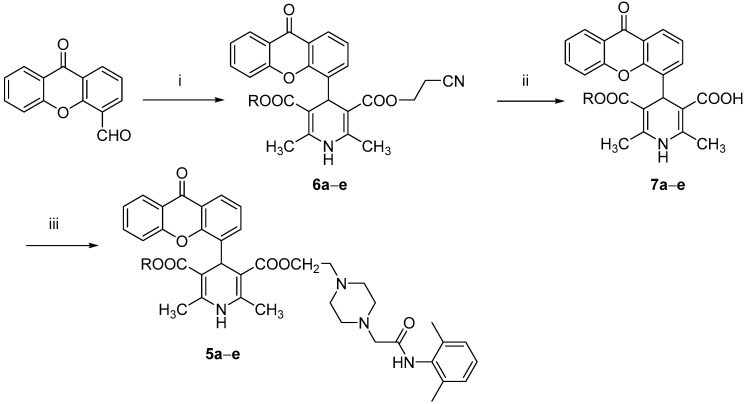
Reagents and conditions: (i) 2-cyanoethyl 3-oxobutanoate, selected aminocrotonate, isopropanol, reflux; 20 h; (ii) LiOH, rt, 16 h; (iii) *N*-(2,6-dimethylphenyl)-2-[4-(2-hydroxy-ethylpiperazin-1-yl)]acetamide, CDI or DCC, THF, 120 °C, 1 h.

All the tested compounds were synthesized applying the Hantszch reaction, starting from xanthen-9-one-4-carboxyaldehyde, prepared from the bromomethyl derivative as previously reported [12]. According to Scheme 1, the 1,4 DHPs **3a**–**e**, prepared as described in our previous papers [12,13], were treated with pyridinium bromide perbromide to afford the corresponding 2-bromomethyl intermediates, which were condensed without further purification with *N*-(2,6-dimethyl-phenyl)-2-piperazin-1-ylacetamide (prepared as described in the literature [18]) to obtain compounds **4a**–**e**. The synthesis of derivatives **5a**–**e** is depicted in Scheme 2. The xanthen-9-one-4-carboxyaldehyde was treated with 2-cyanoethyl 3-oxobutanoate and the selected aminocrotonate to obtain the 1,4-DHP cyanoethyl ester derivatives **6a**–**e**, which were selectively hydrolyzed with LiOH to yield the corresponding monoesters **7a**–**e**, which in turn were condensed with *N*-(2,6-dimethylphenyl)-2-[4-(2-hydroxy-ethylpiperazin-1-yl)]acetamide [19] in tetrahydrofuran (THF) in the presence of 1,1′-carbonyldiimidazole (CDI) or dicyclohexylcarbodiimide (DCC).

### 2.2. Functional Study on Cardiac System

The results are reported in Table 1 (cardiac parameters) and Table S1 (vascular smooth muscle, see Supplementary Materials, SM), together with those of the previously reported Xant-DHPs **3a**–**e**, hybrids nifedipine–lidoflazine 1 and 2, and the reference compounds nifedipine and lidoflazine.

As reported in Table 1 and Table S1 (SM), on isolated guinea-pig tissues, nifedipine showed negative inotropic and chronotropic effects and a well-known vasorelaxant activity. Conversely, lidoflazine, while being almost 30 times less potent than nifedipine, proved to be more selective, only displaying inotropic effects, observed on both the left and right atria. For our hybrid compounds **1** and **2**, the presence of the same 4-*o*-nitrophenyl substituent found in nifedipine led to a similar cardiovascular profile, even if to a different extent. Compound **1**, bearing the fragment of lidoflazine in position 2, showed similar negative chronotropic and inotropic potencies (EC_50_ = 0.72 µM and EC_50_ = 1.01 µM, respectively), although significantly lower than those of nifedipine, together with a slight vasorelaxant activity. On the contrary, for compound **2**, in which the lidoflazine fragment was inserted in position 3, the negative inotropic potency was increased 4.5-fold with respect to nifedipine and the negative chronotropic activity was reduced 4-fold, leading to a reversed selectivity. Notably, compound **2** was also devoid of vasorelaxant activity.

As stated in our previous papers [12,13], the substitution of the 4-*o*-nitrophenyl ring with a 4-Xant moiety in the 1,4-DHP skeleton led to a substantial mutation in the activity profile of compounds with respect to nifedipine, namely an unexpected selectivity for cardiac functional parameters compared to vascular ones. In particular, xanthodipine **3a**, the most structurally similar to the reference compound, and the diethylester analog **3b** only showed negative chronotropic activity, while the introduction in positions 3 and 5 of different ester groups, in particular isopropyl and allyl esters (compounds **3c** and **3d**), allowed obtaining Xant-DHPs endowed with both negative chronotropic and inotropic activities. In contrast, in the new Xant-DHPs **4a**–**e** and **5a**–**e**, the hybridization with lidoflazine led to a different behavior. Compounds **4a** and **5a**, related to xanthodipine, showed remarkable negative inotropic activity, but **4a** alone also exhibited this activity on a spontaneously beating right atrium, and **5a** appeared to be less selective, since a negative chronotropic activity was observed. However, it should be noted that the inotropic effect remained 2.3 times higher than the chronotropic one. In the previously reported series, the Xant-diethylester DHP **3b** showed a more evident chronotropic effect with respect to xanthodipine **3a**. Now, the hybridization with lidoflazine allowed obtaining compounds **4b** and **5b**, endowed with an additional negative inotropic effect and a reduced chronotropic activity. As already mentioned, the Xant-diisopropyl DHP **3c** displayed, besides the chronotropic effects, a consistent negative inotropic effect that was not observed with **3a** and **3b**, but was retained by the corresponding new hybrid derivatives **4c** and **5c**. Compound **5c**, but not **4c**, also maintained the negative chronotropic activity, even if to a lesser extent. The Xant-diallyl DHP **3d** proved to be the least selective of the previous series, showing a pronounced activity on both cardiac parameters. Surprisingly, the hybridization with lidoflazine led to compounds (**4d** and **5d**) devoid of significant activity on the examined parameters, with only compound **5d** showing a weak negative chronotropic potency. The opposite trend can be observed for the propargyl ester compounds: the nonhybrid DHP **3e** was quite inactive, while the new compound **4e** proved to be endowed with a selective negative inotropic activity, and the 3-substituted hybrid **5e** showed the most potent negative inotropic activity among the newly synthesized compounds, together with a remarkable chronotropic potency.

None of the new compounds displayed significant vascular effects (Table S1, SM).

**Figure 3:**
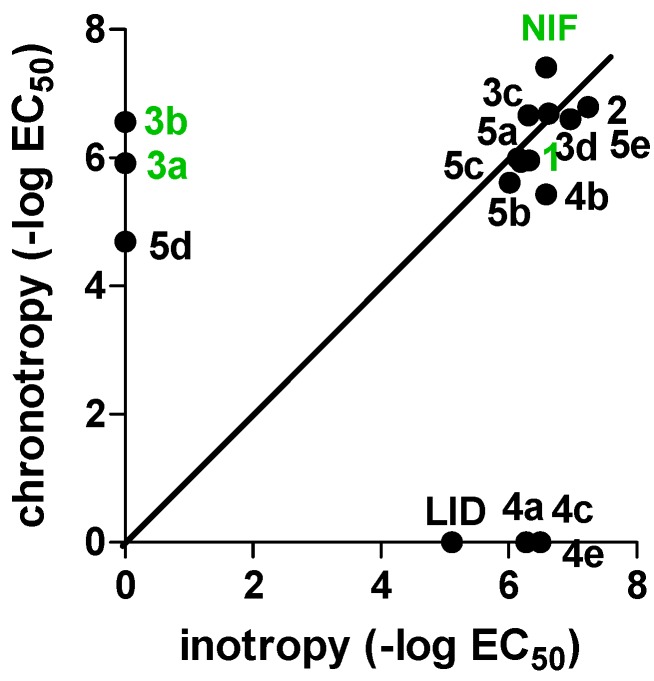
Design strategy for the studied compounds **4a**–**e** and **5a**–**e**.

For the sake of clarity, the results on cardiac parameters are depicted in Figure 3.

## 3. Discussion

Due to the vast array of tolerated structural modifications, the 1,4-DHP scaffold has been widely exploited aiming at developing multifunctional compounds [21]. In particular, our research group reported a series of 4-(xanthen-9-one) DHPs endowed with a selective negative chronotropic activity, which could suggest a possible preferential interaction with the cardiac isoform Ca_v_ 1.3 of the α1 subunit of CC. On the other hand, lidoflazine, an atypical calcium antagonist that also acts in cardiomyocytes as nucleoside transport inhibitor, showed an interesting selective negative inotropic effect in our experimental studies [11]. In this paper, a small series of hybrid compounds was designed, in which the peculiar fragment of lidoflazine was introduced in position 2 or 3 on the 4-Xant-DHP core.

A first evaluation of the functional studies clearly indicates for the new derivatives **4a**–**e** and **5a**–**e** an improved negative inotropic activity with respect to lidoflazine, which seems unrelated to the position of the lidoflazine fragment. Taking a closer look at the results, for derivatives **4a**/**5a**, **4b**/**5b**, and **4e**/**5e**, this effect seems strictly associated to the presence of the lidoflazine fragment, being the corresponding previously reported Xant-DHPs devoid of this activity. In particular, the 3-substituted 1,4-DHP derivative **5e** (bearing a 3-propargyl ester) appears to be the most active of the series and is twofold more potent than nifedipine itself. On the other hand, regarding compounds **4c**/**5c**, which bear an isopropyl ester, negative inotropic effects seemed unrelated to the presence and the position of the lidoflazine fragment, having also been recorded for the parent Xant-DHP **3c**. Remarkably, most of the 2-substituted derivatives (**4a**, **4c**, and **4e**) proved to be selective for negative inotropism, showing a relevant activity also on the spontaneously beating right atrium and being devoid of chronotropic activity. A similar trend could be also observed for **4b**, which despite showing a mixed activity, proved to be tenfold more potent in negative inotropy. Therefore, the introduction of the lidoflazine fragment in position 2 increased the selectivity for cardiac inotropism, leading to speculation about possible preferential binding of **4a**–**e** with the Ca_v_ 1.2 subtype, which is involved in cardiomyocyte contractility. This peculiar behavior could be exploited as tool for an in-depth study of the CC subtypes. Interestingly, among compounds bearing allyl ester groups (**3d**, **4d**, and **5d**), a detectable negative inotropic effect was only observed for the unsubstituted Xant-DHP **3d**, while the new allyl-DHP derivatives **4d** and **5d** were devoid of appreciable activity.

Regarding cardiodepressant activity, a different picture can be drawn, with a more evident influence of the position of the lidoflazine portion. Indeed, when this moiety was inserted in position 3 (**5a**–**e**), a significant activity was observed, with **5e** again being the most effective compound, with a cardiodepressant activity comparable to that of the unsubstituted Xant-DHPs **3a**–**e**. As previously reported, for the 2-substituted derivatives **4a**–**e**, a poor chronotropic effect was detected.

As far as calcium antagonism is concerned, all the tested compounds proved to be only weakly active, with only xanthodipine **3a** and the diethyl derivative **3b** showing significant IC_50s_, but still much lower than that of nifedipine.

In summary, in this paper, a series of Xant-lidoflazine 1,4-DHP hybrid compounds was synthesized and evaluated to investigate their cardiovascular profiles. The functional studies indicated an interesting peculiar selectivity for the cardiac parameter inotropy, in particular when the lidoflazine fragment was introduced in position 2 of the DHP scaffold, usually substituted with small groups. With respect to our previously reported Xant-based 1,4-DHPs (**3a**–**e**), the new modifications led to an inversion of selectivity, from the negative chronotropic **3a**–**e** to the negative inotropic **4a**–**e**. This trend could suggest a preferential binding of the compounds to different LTCC Ca_v_ 1 isoforms: Ca_v_ 1.3, responsible for chronotropy, for Xant-DHPs **3a**–**e**; and Ca_v_ 1.2, mainly involved in cardiac contractility, for the mixed Xant-lidoflazine 1,4-DHPs **4a**–**e**. Considering that the development of isoform-selective ligands could represent a step forward in the treatment of various diseases, and taking into account the relatively weak potency on cardiovascular tissues, these compounds can be seen as potential tools to study different subtypes of CCs. Further studies could indeed be performed in order to improve the drug-likeness of these compounds.

## 4. Materials and Methods

### 4.1. Chemistry

#### 4.1.1. General Methods

Starting materials used, unless otherwise specified, were high-grade commercial products. Solvents were of analytical grade. Reaction progress was followed by thin layer chromatography (TLC) on precoated silica gel plates (Merck Silica Gel 60 F254, Merck, Darmstadt, Germany) and then visualized with a UV254 lamplight. Chromatographic separations were performed on silica gel columns by the flash method (Kieselgel 40, 0.040–0.063 mm, Merck, Darmstadt, Germany). Melting points were determined in open glass capillaries using a Büchi apparatus, and are uncorrected. ^1^H NMR and ^13^C NMR spectra were recorded on a Varian Gemini spectrometer at 400 MHz and 101 MHz, respectively, in CDCl_3_ solutions unless otherwise indicated, and chemical shifts (δ) were reported as parts per million (ppm) values relative to tetramethylsilane (TMS) as the internal standard; coupling constants (*J*) are reported in Hertz (Hz). Standard abbreviations indicating spin multiplicities are given as follows: s (singlet), d (doublet), t (triplet), br (broad), q (quartet), or m (multiplet). Mass spectra were recorded on a Waters ZQ 4000 apparatus operating in electrospray mode (ES). Chemical purities of tested compounds were determined by elemental analysis (C, H, N) and were within ±0.4% of the theoretical values. Compounds were named relying on the naming algorithm developed by CambridgeSoft Corporation (Waltham, MA, USA) and used in ChemDraw Professional 15.0 (Perkin Elmer INC., Waltham, MA, USA).

#### 4.1.2. General Method I: Preparation of **4a**–**e**

The selected 4-Xant-dihydropyridine (**3a**–**e**) [12,13] (2.7 mmol) in CH_2_Cl_2_ was cooled at 0 °C and treated with 0.31 mL of pyridine and 0.93 g of pyridinium bromide perbromide (2.9 mmol). The mixture was stirred at 0 °C for 40 min, diluted with CH_2_Cl_2_ (30 mL), and washed with HCl 2N (3 × 20 mL) and brine. The organic layer was evaporated to dryness and the residue was solubilized in THF (20 mL). A suspension of *N*-(2,6-dimethyl-phenyl)-2-piperazin-1-ylacetamide (5.4 mmol) and K_2_CO_3_ (1 g) in DMF was then slowly added and the resulting mixture was stirred at room temperature overnight. The solvent was concentrated under reduced pressure, and the residue was diluted with CH_2_Cl_2_ (30 mL), washed with H_2_O (3 × 30 mL), dried over sodium sulfate, and the solvent evaporated to dryness.

*Dimethyl 2-((4-(2-((2,6-Dimethylphenyl)amino)-2-oxoethyl)piperazin-1-yl)methyl)-6-methyl-4-(9-oxo-9H-xanthen-4-yl)-1,4-dihydropyridine-3,5-dicarboxylate* (**4a**). The title compound was obtained starting from **3a** (30%) and was purified by flash column chromatography (toluene/acetone 3/2) and crystallization from toluene; mp 110–112 °C. ^1^H NMR δ: 2.23 (s, 6H), 2.44 (s, 3H), 2.68 (broad, 4H), 2.81 (broad, 4H), 3.28 (s, 2H), 3.52 (s, 3H), 3.53 (s, 3H), 3.74–3.91 (m, 2H), 5.70 (s, 1H), 7.10–7.12 (m, 3H arom), 7.39 (t, *J* = 8.0 Hz, 1H arom), 7.52 (d, *J* = 8.0, 1H arom), 7.68–7.74 (m, 2H arom), 7.99 (s, 1H, NH DHP), 8.20 (dd, *J* = 8.0 and 1.6 Hz, 1H arom), 8.35 (dd, *J* = 8.0 and 1.6 Hz, 1H arom), 8.54 (s, 1H, CONH). ^13^C NMR δ: 18.6, 19.7, 35.8, 35.9, 50.9, 53.5, 53.9, 56.6, 61.7, 102.2, 102.9, 111.2, 117.5, 121.8, 123.4, 123.7, 125.2, 126.9, 127.3, 128.3, 129.0, 131.5, 133.4, 134.6, 134.8, 135.5, 136.5, 144.7, 153.4, 155.3, 156.1, 164.0, 166.7, 167.5, 167.9, 168.3, 177.5. ES-MS: *m*/*z* 661 [M + H].

*Diethyl 2-((4-(2-((2,6-Dimethylphenyl)amino)-2-oxoethyl)piperazin-1-yl)methyl)-6-methyl-4-(9-oxo-9H-xanthen-4-yl)-1,4-dihydropyridine-3,5-dicarboxylate* (**4b**). The title compound was obtained starting from **3b** (33%) and was purified by flash column chromatography (toluene/acetone 3/2) and crystallization from toluene; mp 234–236 °C. ^1^H NMR δ: 0.97–1.01 (m, 6H), 2.23 (s, 6H), 2.43 (s, 3H), 2.69 (broad, 4H), 2.81 (broad, 4H), 3.28 (s, 2H), 3.76–3.99 (m, 6H), 5.70 (s, 1H), 7.08–7.12 (m, 3H arom), 7.28–7.30 (m, 1H arom), 7.38 (t, J = 8.0 Hz, 1H arom), 7.50 (d, J = 8.0, 1H arom), 7.71–7.75 (m, 2H arom), 7.99 (s, 1H, NH DHP), 8.21 (dd, J = 8.0 and 1.6 Hz, 1H arom), 8.36 (dd, J = 8.0 and 1.6 Hz, 1H arom), 8.55 (s, 1H, CONH). ^13^C NMR δ: 15.2, 18.6, 19.7, 35.8, 35.9, 50.9, 53.5, 53.9, 56.5, 61.7, 102.2, 102.9, 111.2, 117.5, 121.8, 123.4, 123.7, 125.2, 126.9, 127.3, 128.3, 129.0, 131.5, 133.4, 134.5, 134.8, 135.4, 136.5, 144.7, 153.4, 155.3, 156.2, 164.1, 166.7, 167.5, 168.0, 168.4, 177.5. ES-MS: *m*/*z* 693 [M + H].

*Diisopropyl 2-((4-(2-((2,6-Dimethylphenyl)amino)-2-oxoethyl)piperazin-1-yl)methyl)-6-methyl-4-(9-oxo-9H-xanthen-4-yl)-1,4-dihydropyridine-3,5-dicarboxylate* (**4c**). The title compound was obtained starting from **3c** (28%) and was purified by flash column chromatography (toluene/acetone 3/2) and crystallization from toluene; mp 134–136 °C. ^1^H NMR δ: 0.68–0.72 (m, 6H), 1.12–1.18 (m, 6H), 2.24 (s, 6H), 2.42 (s, 3H), 2.69 (broad, 4H), 2.81 (broad, 4H), 3.27 (s, 2H), 3.75–3.96 (m, 2H), 4.81–4.93 (m, 2H), 5.68 (s, 1H), 7.11–7.15 (m, 3H arom), 7.28–7.30 (m, 1H arom), 7.38 (t, J = 8.0 Hz, 1H arom), 7.50 (d, J = 8.0, 1H arom), 7.73–7.77 (m, 2H arom), 7.97 (s, 1H, NH DHP), 8.20 (dd, J = 8.0 and 1.6 Hz, 1H arom), 8.37 (dd, J = 8.0 and 1.6 Hz, 1H arom), 8.58 (s, 1H, CONH). ^13^C NMR δ: 18.6, 19.7, 21.7, 36.0, 53.5, 54.0, 56.5, 61.7, 68.8, 102.3, 102.9, 111.2, 117.5, 121.8, 123.4, 123.7, 125.2, 126.9, 127.2, 128.3, 129.0, 131.5, 133.4, 134.5, 134.9, 135.4, 136.5, 144.7, 153.4, 155.3, 156.2, 164.1, 166.7, 167.5, 168.0, 168.3, 177.5. ES-MS: *m*/*z* 721 [M + H].

*Diallyl 2-((4-(2-((2,6-Dimethylphenyl)amino)-2-oxoethyl)piperazin-1-yl)methyl)-6-methyl-4-(9-oxo-9H-xanthen-4-yl)-1,4-dihydropyridine-3,5-dicarboxylate* (**4d**). The title compound was obtained starting from **3d** (28%) and was purified by flash column chromatography (toluene/acetone 3/2) and crystallization from toluene; mp 112–115 °C. ^1^H NMR δ 2.23 (s, 6H), 2.44 (s, 3H), 2.69 (broad, 4H), 2.82 (broad, 4H), 3.28 (s, 2H), 3.76–3.94 (m, 2H), 4.38–4.41 (m, 4H), 4.86–4.96 (m, 4H), 5.54–5.60 (m, 2H), 5.77 (s, 1H), 7.09–7.12 (m, 3H arom), 7.29–7.31 (m, 1H arom), 7.38 (t, *J* = 8.0 Hz, 1H arom),7.47 (d, *J* = 8.0, 1H arom), 7.70–7.72 (m, 2H arom), 8.00 (s, 1H, NH DHP), 8.18 (dd, *J* = 8.0 and 1.6 Hz, 1H arom), 8.34 (dd, *J* = 8.0 and 1.6 Hz, 1H arom), 8.55 (s, 1H, CONH). ^13^C NMR δ: 18.6, 19.8, 35.3, 53.5, 53.9, 56.6, 61.7, 64.5, 64.6, 102.47, 103.2, 117.7, 117,9, 121.5, 121.6, 123.4, 123.6, 125.2, 126.7, 127.3, 128.3, 132.3, 132.4, 133.4, 134.3, 134.8, 136.4, 137.3, 144.9, 145.1, 153.1, 156.1, 166.7, 166.9, 168.0, 177.6. ES-MS: *m*/*z* 718 [M + H].

*Di(prop-2-yn-1-yl) 2-((4-(2-((2,6-Dimethylphenyl)amino)-2-oxoethyl)piperazin-1-yl)methyl)-6-methyl-4-(9-oxo-9H-xanthen-4-yl)-1,4-dihydropyridine-3,5-dicarboxylate* (**4e**). The title compound was obtained starting from **3e** (28%) and was purified by flash column chromatography (toluene/acetone 3/2) and crystallization from toluene; mp 171–174 °C. ^1^H NMR δ: 2.02 (t, *J* = 2.4 Hz, 2H), 2.23 (s, 6H), 2.45 (s, 3H), 2.70 (broad, 4H), 2.82 (broad, 4H), 3.28 (s, 2H), 3.76–3.93 (m, 2H), 4.89–4.51 (m, 4H), 5.78 (s, 1H), 7.08–7.12 (m, 3H arom), 7.29–7.31 (m, 1H arom), 7.37 (t, *J* = 7.6 Hz, 1H arom),7.56 (d, *J* = 8.8, 1H arom), 7.70–7.74 (m, 2H arom), 8.11 (s, 1H, NH DHP), 8.21 (dd, *J* = 8.4 and 1.6 Hz, 1H arom), 8.35 (dd, *J* = 8.0 and 1.6 Hz, 1H arom), 8.54 (s, 1H, CONH). ^13^C NMR δ: 18.6, 19.8, 35.0, 51.2, 53.5, 53.9, 56.6, 61.7, 74.1, 74.2, 102.9, 118.2, 121.6, 121.7, 123.5, 125.3, 126.6, 127.3, 128.3, 133.4, 134.3, 134.8, 136.3, 136.9, 145.5, 145.7, 156.3, 166.1, 166.3, 168.0, 177.6. ES-MS: *m*/*z* 713 [M + H].

#### 4.1.3. General Method II: Preparation of **6a**–**e**

A solution of 9-oxo-9H-xanthene-4-carbaldehyde (1 eq), 2-cyanoethyl 3-oxobutanoate (1 eq), and the selected aminocrotonate (1 eq) in isopropanol was heated under reflux for 20 h. The solvent was removed under reduced pressure and the residue was purified by flash column chromatography (toluene/ethyl acetate 9/1).

*3-(2-Cyanoethyl) 5-methyl 2,6-dimethyl-4-(9-oxo-9H-xanthen-4-yl)-1,4-dihydropyridine-3,5-dicarboxylate* (**6a**). The compound (50%) was obtained starting from methyl 3-aminocrotonate. Mp 212–214 °C. ^1^H NMR δ: 2.40 (s, 3H), 2.43 (s, 3H), 2.46–2.59 (m, 2H), 3.55 (s, 3H), 4.08–4.28 (m, 2H), 5.67 (s, 1H), 5.94 (s, 1H, NH DHP), 7.31 (t, *J* = 7.2 Hz, 1H arom), 7.42 (t, *J* = 7.2 Hz, 1H arom), 7.63 (d, *J* = 8.0 Hz, 1H arom), 7.75–7.79 (m, 2H arom), 8.26 (d, *J* = 7.4 Hz, 1H arom), 8.39 (d, *J* = 7.4 Hz, 1H arom).

*3-(2-cyanoethyl) 5-ethyl 2,6-dimethyl-4-(9-oxo-9H-xanthen-4-yl)-1,4-dihydropyridine-3,5-dicarboxylate* (**6b**). The compound (60%) was obtained starting from ethyl 3-aminocrotonate. Mp 204–206 °C. ^1^H NMR δ: 0.99 (t, *J* = 8.0 Hz, 3H), 2.37 (s, 3H), 2.39 (s, 3H), 2.46–2.55 (m, 2H), 3.92–3.96 (m, 2H), 4.08–4.29 (m, 2H), 5.67 (s, 1H), 6.20 (s, 1H, NH DHP), 7.29 (t, *J* = 7.2 Hz, 1H arom), 7.41 (t, *J* = 7.2 Hz, 1H arom), 7.62 (d, *J* = 7.6 Hz, 1H arom), 7.74–7.77 (m, 2H arom), 8.23 (d, *J* = 7.2 Hz, 1H arom), 8.33 (d, *J* = 7.2 Hz,1H arom).

*3-(2-Cyanoethyl) 5-isopropyl 2,6-dimethyl-4-(9-oxo-9H-xanthen-4-yl)-1,4-dihydropyridine-3,5-dicarboxylate* (**6c**). The compound (55%) was obtained starting from isopropyl 3-aminocrotonate [22]. Mp 186–188 °C. ^1^H NMR δ: 0.78 (d, *J* = 6.0 Hz, 3H), 1.17 (d, *J* = 6.0 Hz, 3H), 2.40 (s, 3H), 2.42 (s, 3H), 2.50–2.54 (m, 2H), 4.08–4.28 (m, 2H), 4.83–4.90 (m, 1H), 5.68 (s, 1H), 6.08 (s, 1H, NH DHP), 7.31–7.42 (m, 2H arom), 7.64 (d, *J* = 8.0 Hz, 1H arom), 7.76–7.80 (m, 2H arom), 8.26 (d, *J* = 7.4 Hz, 1H arom), 8.38 (d, *J* = 7.4 Hz, 1H arom).

*3-(2-Cyanoethyl) 5-allyl 2,6-dimethyl-4-(9-oxo-9H-xanthen-4-yl)-1,4-dihydropyridine-3,5-dicarboxylate* (**6d**). The compound (58%) was obtained starting from allyl 3-aminocrotonate [23]. Mp 190–192 °C. ^1^H NMR δ: 2.39 (s, 3H), 2.41 (s, 3H), 2.44–2.53 (m, 2H), 3.98–4.28 (m, 2H), 4.40 (d, *J* = 6.0 Hz, 2H), 4.88–4.96 (m, 2H), 5.52–5.66 (m, 1H), 5.70 (s, 1H), 6.05 (s, 1H, NH DHP), 7.29–7.42 (m, 2H arom), 7.67 (d, *J* = 8.0 Hz, 1H arom), 7.76–7.78 (m, 2H arom), 8.23 (d, *J* = 7.4 Hz, 1H arom), 8.39 (d, *J* = 7.4 Hz, 1H arom).

*3-(2-Cyanoethyl) 5-propargyl 2,6-dimethyl-4-(9-oxo-9H-xanthen-4-yl)-1,4-dihydropyridine-3,5-dicarboxylate* (**6e**). The compound (62%) was obtained starting from propargyl 3-aminocrotonate [22]. Mp 272–275 °C. ^1^H NMR δ: 2.01 (t, *J* = 3.0 Hz, 1H), 2.39 (s, 3H), 2.42 (s, 3H), 2.46–2.51 (m, 2H), 3.98–4.28 (m, 2H), 4.50 (s, 2H), 5.70 (s, 1H), 6.28 (s, 1H, NH DHP), 7.28–7.45 (m, 2H arom), 7.67 (d, *J* = 8.0 Hz, 1H arom), 7.73–7.77 (m, 2H arom), 8.23 (d, *J* = 7.4 Hz, 1H arom), 8.39 (d, *J* = 7.4 Hz, 1H arom).

#### 4.1.4. General Method III: Preparation of Monoesters **7a**–**e**

Dihydropyridine derivatives **6a**–**e** (2.8 mmol) were dissolved in ethanol and LiOH (5.0 mmol) was added. The solution was stirred at rt for 16 h, the solvent was removed in vacuo, and the residue was kept in H_2_O, filtered, and acidified with diluted (1:1) HCl. Derivatives **7a**–**e** were obtained by filtration and used in the subsequent step without further purification.

*5-(Methoxycarbonyl)-2,6-dimethyl-4-(9-oxo-9H-xanthen-4-yl)-1,4-dihydropyridine-3-carboxylic acid* (**7a**). The compound was obtained starting from **6a** (72%). Mp 197–199 °C. ^1^H NMR δ: 2.40 (s, 3H), 2.43 (s, 3H), 3.55 (s, 3H), 5.69 (s, 1H), 5.98 (s, 1H, NH DHP), 7.30 (t, *J* = 7.2 Hz, 1H arom), 7.42 (t, *J* = 7.2 Hz, 1H arom), 7.65 (d, *J* = 8.0 Hz, 1H arom), 7.75–7.80 (m, 2H arom), 8.27 (d, *J* = 7.4 Hz, 1H arom), 8.39 (d, *J* = 7.4 Hz, 1H arom).

*5-(Ethoxycarbonyl)-2,6-dimethyl-4-(9-oxo-9H-xanthen-4-yl)-1,4-dihydropyridine-3-carboxylic acid* (**7b**). The compound was obtained starting from **6b** (70%). Mp 190–193 °C. ^1^H NMR (DMSO) δ: 0.98 (t, *J* = 8.0 Hz, 3H), 2.27 (s, 3H), 2.29 (s, 3H), 3.80 (q, *J* = 8.0 Hz, 2H), 5.48 (s, 1H), 7.31 (t, *J* = 7.2 Hz, 1H arom), 7.48 (t, *J* = 7.2 Hz, 1H arom), 7.68–7.72 (m, 2H arom), 7.81–7.92 (m, 1H arom), 8.01 (d, *J* = 7.2 Hz, 1H arom), 8.18 (d, *J* = 7.2 Hz, 1H arom), 9.02 (s, 1H, NH DHP).

*5-(Isopropoxycarbonyl)-2,6-dimethyl-4-(9-oxo-9H-xanthen-4-yl)-1,4-dihydropyridine-3-carboxylic acid* (**7c**). The compound was obtained starting from **6c** (65%). Mp 157–160 °C. ^1^H NMR δ: 0.81 (d, *J* = 6.0 Hz, 3H), 1.14 (d, *J* = 6.0 Hz, 3H), 2.30 (s, 3H), 2.36 (s, 3H), 4.83–4.91 (m, 1H), 5.55 (s, 1H), 6.08 (s, 1H, NH DHP), 7.20–7.38 (m, 2H arom), 7.48 (d, *J* = 8.0 Hz, 1H arom), 7.65–7.76 (m, 2H arom), 8.12 (d, *J* = 7.4 Hz, 1H arom), 8.28 (d, *J* = 7.4 Hz, 1H arom).

*5-((Allyloxy)carbonyl)-2,6-dimethyl-4-(9-oxo-9H-xanthen-4-yl)-1,4-dihydropyridine-3-carboxylic acid* (**7d**). The compound was obtained starting from **6d** (69%). Mp 177–179 °C. ^1^H NMR (DMSO) δ: 2.329 (s, 3H), 2.32 (s, 3H), 4.34 (d, *J* = 6.0 Hz, 2H), 4.82–4.93 (m, 2H), 5.41 (s, 1H), 5.52–5.68 (m, 1H), 7.28–7.42 (m, 2H arom), 7.63–7.69 (m, 2H arom), 7.76–7.80 (m, 2H arom), 8.16 (d, *J* = 7.4 Hz, 1H arom), 9.05 (s, 1H, NH DHP).

*2,6-Dimethyl-4-(9-oxo-9H-xanthen-4-yl)-5-((prop-2-yn-1-yloxy)carbonyl)-1,4-dihydropyridine-3-carboxylic acid* (**7e**). The compound was obtained starting from **6e** (60%). Mp 139–141 °C. ^1^H NMR δ: 2.00 (t, *J* = 3.0 Hz, 1H), 2.39 (s, 3H), 2.42 (s, 3H), 4.53 (s, 2H), 5.60 (s, 1H), 6.28 (s, 1H, NH DHP), 7.28–7.45 (m, 2H arom), 7.68 (d, *J* = 8.0 Hz, 1H arom), 7.73–7.79 (m, 2H arom), 8.19 (d, *J* = 7.4 Hz, 1H arom), 8.27 (d, *J* = 7.4 Hz, 1H arom).

#### 4.1.5. General Method IV: Preparation of Compounds **5a**–**e**

To a solution of 1.77 mmol of the previously obtained monoester in THF (7.5 mL), 0.5 g of CDI (3.5 mmol) were added and the slurry was stirred for 1 h at rt. The solvent was removed under reduced pressure, *N*-(2,6-dimethylphenyl)-2-(4-(2-hydroxyethyl)piperazin-1-yl)acetamide (1.75 mmol) was added, and the mixture was heated at 120 °C for 50 min. After cooling, the mixture was purified by flash column chromatography (toluene/acetone 1/1).

*3-(2-(4-(2-((2,6-Dimethylphenyl)amino)-2-oxoethyl)piperazin-1-yl)ethyl) 5-methyl 2,6-dimethyl-4-(9-oxo-9H-xanthen-4-yl)-1,4-dihydropyridine-3,5-dicarboxylate* (**5a**). The compound was obtained starting from **7a** (40%). Mp 254–256 °C (toluene). ^1^H NMR δ: 2.25 (s, 6H), 2.27–2.34 (m, 6H), 2.36 (s, 3H), 2.39 (s, 3H), 2.53 (s, 4H), 3.12 (s, 2H), 3.51 (s, 3H), 3.97–4.12 (m, 2H), 5.69 (s, 1H), 5.83 (s, 1H, NH DHP), 7.10–7.12 (m, 3H arom), 7.28–7.30 (m, 1H arom), 7.36 (t, *J* = 7.2 Hz, 1H arom), 7.57 (d, *J* = 7.6, 1H, arom), 7.72–7.76 (m, 2H arom), 8.18 (dd *J* = 8.4 and 1.6 Hz, 1H arom), 8.33 (d, *J* = 6.4 Hz, 1H arom), 8.57 (s, 1H, NHCO). ^13^C NMR δ: 18.6, 19.5, 19.6, 35.0, 50.9, 53.1, 53.6, 56.3, 60.8, 61.6, 102.9, 103.2, 117.7, 121.6, 121.6, 123.5, 123.7, 125.1, 126.8, 127.2, 128.2, 133.6, 134.7, 134.9, 136.2, 137.1, 144.3, 145.0, 153.1, 156.1, 167.1, 167.8, 168.4, 177.5. ES-MS: *m/z* 679 [M + H].

*3-(2-(4-(2-((2,6-Dimethylphenyl)amino)-2-oxoethyl)piperazin-1-yl)ethyl) 5-ethyl 2,6-dimethyl-4-(9-oxo-9H-xanthen-4-yl)-1,4-dihydropyridine-3,5-dicarboxylate* (**5b**). The compound was obtained starting from **7b** (35%). Mp 206–210 °C (toluene). ^1^H NMR δ: 1.10 (t, *J* = 8.0 Hz, 3H), 2.25 (s, 6H), 2.27–2.34 (m, 6H), 2.36 (s, 3H), 2.39 (s, 3H), 2.57 (s, 4H), 3.15 (s, 2H), 3.51 (s, 3H), 3.92–4.22 (m, 4H), 5.68 (s, 1H), 5.90 (s, 1H, NH DHP), 7.12–7.15 (m, 3H arom), 7.28–7.31 (m, 1H arom), 7.36 (t, *J* = 7.2 Hz, 1H arom), 7.56 (d, *J* = 7.6, 1H, arom), 7.73–7.78 (m, 2H arom), 8.18 (dd *J* = 8.4 and 1.6 Hz, 1H arom), 8.32 (d, *J* = 6.4 Hz, 1H arom), 8.57 (s, 1H, NHCO). ^13^C NMR δ: 15.3, 18.6, 19.6, 19.6, 35.1, 50.9, 53.6, 56.3, 60.8, 61.6, 63.4, 103.0, 103.3, 117.7, 121.6, 121.6, 123.5, 123.7, 125.1, 126.9, 127.1, 128.2, 133.6, 134.7, 135.0, 136.2, 137.1, 144.3, 145.0, 153.1, 156.1, 167.1, 167.8, 168.4, 177.5. ES-MS: *m/z* 693 [M + H].

*3-(2-(4-(2-((2,6-Dimethylphenyl)amino)-2-oxoethyl)piperazin-1-yl)ethyl) 5-isopropyl 2,6-dimethyl-4-(9-oxo-9H-xanthen-4-yl)-1,4-dihydropyridine-3,5-dicarboxylate* (**5c**). The compound was obtained starting from **7c** (40%). Mp 206–210 °C (toluene). ^1^H NMR δ: 0.76 (d, *J* = 6.0 Hz, 3H), 1.10 (d, *J* = 6.0 Hz, 3H), 2.23 (s, 6H), 2.24–2.29 (m, 4H), 2.32–2.35 (m, 2H), 2.38 (s, 6H), 2.55 (broad, 4H), 3.13 (s, 2H), 3.96–4.14 (m, 2H), 4.86–4.91 (m, 1H), 5.65 (s, 1H), 5.71 (s, 1H, NH DHP), 7.08–7.11 (m, 3H arom), 7.28–7.30 (m, 1H arom), 7.37 (t, *J* = 7.2 Hz, 1H arom), 7.57 (d, *J* = 8.4 Hz, 1H, arom), 7.73–7.78 (m, 2H arom), 8.17 (dd *J* = 8.0 and 1.6 Hz, 1H arom), 8.32 (d, *J* = 6.8 Hz, 1H arom), 8.57 (s, 1H, NHCO). ^13^C NMR δ: 18.7, 19.5, 19.6, 21.4, 35.1, 53.1, 53.6, 56.3, 60.8, 61.6, 65.4, 103.0, 103.2, 117.7, 121.6, 121.6, 123.5, 123.6, 125.1, 126.8, 127.1, 128.2, 133.6, 134.7, 134.9, 136.2, 137.1, 144.3, 145.0, 153.1, 156.2, 167.1, 167.8, 168.4, 177.5. ES-MS: *m*/*z* 707 [M + H].

*3-Allyl 5-(2-(4-(2-((2,6-dimethylphenyl)amino)-2-oxoethyl)piperazin-1-yl)ethyl) 2,6-dimethyl-4-(9-oxo-9H-xanthen-4-yl)-1,4-dihydropyridine-3,5-dicarboxylate* (**5d**). The compound was obtained starting from **7d** (30%). Mp 82–85 °C (toluene). ^1^H NMR δ: 2.22 (s, 6H), 2.23–2.28 (m, 6H), 2.36 (s, 3H), 2.39 (s, 3H), 2.53 (broad, 4H), 3.12 (s, 2H), 3.97–4.12 (m, 2H), 4.40 (d, *J* = 6.0 Hz, 2H), 4.90–4.96 (m, 2H), 5.57–5.64 (m, 1H), 5.70 (s, 1H), 6.52 (broad, 1H, NH DHP), 7.10–7.14 (m, 3H arom), 7.28–7.30 (m, 1H arom), 7.35 (t, *J* = 7.2 Hz, 1H arom), 7.56 (d, *J* = 7.6, 1H, arom), 7.68–7.73 (m, 2H arom), 8.17 (dd *J* = 8.0 and 1.6 Hz, 1H arom), 8.31 (d, *J* = 6.8 Hz, 1H arom), 8.61 (s, 1H, NHCO). ^13^C NMR δ: 18.5, 19.3, 19.4, 35.2, 53.0, 53.6, 56.3, 60.8, 61.5, 64.5, 102.6, 102.6, 117.7, 118.0, 121.5, 121.6, 121.8, 123.4, 123.6, 124.9, 126.6, 127.4, 128.3, 132.5, 133.4, 134.6, 134.9, 135.0, 136.6, 137.4, 145.4, 145.6, 153.2, 156.1, 167.1, 167.3, 168.9, 177.7. ES-MS: *m*/*z* 705 [M + H].

*3-(2-(4-(2-((2,6-Dimethylphenyl)amino)-2-oxoethyl)piperazin-1-yl)ethyl) 5-(prop-2-yn-1-yl) 2,6-dimethyl-4-(9-oxo-9H-xanthen-4-yl)-1,4-dihydropyridine-3,5-dicarboxylate* (**5e**). The compound was obtained starting from **7e** (30%). Mp 127–129 °C (toluene/petroleum ether 3/2). ^1^H NMR δ: 2.01 (t, *J* = 3.0 Hz, 1H), 2.24 (s, 6H), 2.27–2.34 (m, 6H), 2.36 (s, 3H), 2.38 (s, 3H), 2.53 (s, 4H), 3.12 (s, 2H), 3.98–4.13 (m, 2H), 4.46 (s, 2H), 5.62 (s, 1H), 5.80 (broad, 1H, NH DHP), 7.11–7.13 (m, 3H arom), 7.28–7.30 (m, 1H arom), 7.37 (t, *J* = 7.2 Hz, 1H arom), 7.58 (d, *J* = 7.6, 1H, arom), 7.72–7.76 (m, 2H arom), 8.17 (dd *J* = 8.0 and 1.6 Hz, 1H arom), 8.34 (d, *J* = 6.8 Hz, 1H arom), 8.57 (s, 1H, NHCO). ^13^C NMR δ: 18.6, 19.6, 19.7, 35.0, 51.4, 53.1, 53.6, 56.3, 60.7, 61.6, 72.4, 72.7, 103.0, 103.2, 117.8, 121.6, 121.6, 123.5, 123.7, 125.0, 126.8, 127.1, 128.2, 133.6, 134.7, 134.9, 136.2, 137.1, 144.3, 145.0, 153.1, 156.1, 167.1, 167.8, 168.4, 177.5. ES-MS: *m*/*z* 703 [M + H].

### 4.2. Biology

#### 4.2.1. Animals 

Guinea pigs of either sex (200–400 g) obtained from Charles River (Como, Italy) were used. The animals were housed according to the ECC Council Directive regarding the protection of animals used for experimental and other scientific purposes (Directive 2010/63/EU of the European Parliament and of the Council) and the WMA Statement on Animal Use in Biomedical Research. All procedures followed the guidelines of animal care and use of the University of Bologna and were authorized by the Comitato Etico Scientifico for Animal Research, ID: 737/2017.

#### 4.2.2. Functional Studies

The biological profiles of the new hybrid molecules **4a**–**e** and **5a**–**e** were evaluated on isolated guinea-pig left and right atria to assess their inotropic and/or chronotropic effects, respectively, and on K^+^-depolarized (80 mM) guinea-pig vascular aortic strips to assess calcium antagonist activity. The method used was previously described [24]. Briefly, compounds were checked at increasing doses to evaluate the percent decrease of developed tension on an isolated left atrium driven at 1 Hz (negative inotropic activity) and the percent decrease in atrial rate on a spontaneously beating right atrium (negative chronotropic activity). For compounds devoid of negative chronotropic activity, the inotropy was also checked in a spontaneously beating right atrium. The percent inhibition of calcium-induced contraction was evaluated on K^+^-depolarized smooth muscle strips. Data were analyzed using the Student’s *t*-test and are presented as the mean ±SEM [20]. Since the drugs were added in a cumulative manner, the differences between the control and the experimental values at each concentration were tested for a *p* value of <0.05. The potencies of drugs, defined as the EC_50_ and IC_50_, were evaluated from log concentration–response curves (Probit analysis using Litchfield and Wilcoxon [20] or GraphPad Prism^®^ software [25,26] for the appropriate pharmacological preparations.

## Figures and Tables

**Table 1:** Cardiac activity of the tested compounds.

	Left Atrium			Right Atrium					
	Negative Inotropy			Negative Inotropy			Negative Chronotropy		
Cpd	Activity *^d^*	EC_50_ *^e^*(µM)	SEM	Activity *^f^*	EC_50_ *^e^*(µM)	SEM	Activity *^g^*	EC_50_ *^e^*(µM)	SEM
**Nif**	97 ± 2.0 *^h^*	0.26	0.19–0.36				85 ± 4.2 *^k^*	0.039	0.031–0.051
**Lidofl**	65 ± 5.8	7.71	6.82–8.84	61 ± 1.7 *^j^*	0.31	0.23–0.41	38 ± 1.5		
**1 *^a^***	**59 ± 0.5**	0.72	0.67–0.79				**89 ± 3.4** *^j^*	1.01	0.78–1.30
**2 *^a^***	**96 ± 2.3**	0.058	0.052–0.070				**77 ± 2.5** *^i^*	0.16	0.11–0.23
**3a *^b^***	30 ± 1.7 *^h^*						71 ± 3.1 *^j^*	**1.20**	**0.73–1.53**
**4a**	**62 ± 3.4**	**0.54**	**0.35–0.77**	**64 ± 1.7** *^k^*	**1.15**	**0.87–1.83**	**37 ± 2.3** *^m^*		
**5a**	**62 ± 3.7** *^h^*	**0.48**	**0.41–0.58**				**85 ± 1.3**	**1.09**	**0.89–1.22**
**3b *^b^***	26 ± 2.3 *^h^*						93 ± 2.1 *^i^*	**0.27**	**0.13–0.71**
**4b**	**79 ± 2.4** *^h^*	**0.26**	**0.21–0.34**				**61 ± 3.4**	**3.67**	**2.98–3.88**
**5b**	**55 ± 3.1**	**0.97**	**0.54–1.55**				**88 ± 3.6**	**2.45**	**2.24–3.01**
**3c *^c^***	65 ± 2.4	0.50	0.20–0.80				94 ± 4.6 *^j^*	**0.22**	**0.075–0.58**
**4c**	**67 ± 3.4** *^h^*	**0.32**	**0.26–0.40**	**58 ± 2.4**	**1.62**	**1.06–2.46**	**38 ± 1.2** *^m^*		
**5c**	**78 ± 1.1**	**0.64**	**0.47–0.86**				**86 ± 2.2**	**1.15**	**0.89–1.32**
**3d *^c^***	61 ± 3.6	0.24	0.17–0.30				89 ± 4.4 *^j^*	**0.20**	**0.14–0.28**
**4d**	**34 ± 1.6**			**10 ± 0.7**			**32 ± 2.4**		
**5d**	**44 ± 0.1**						**55 ± 0.5**	**20.15**	**19.02–21.48**
**3e *^c^***	35 ± 2.9			**47 ± 1.4**			34 ± 2.1 *^j^*		
**4e**	**80 ± 2.4** *^i^*	**0.32**	**0.26–0.40**	**19 ± 0.3**			**7 ± 0.3** *^m^*		
**5e**	**88 ± 1.6** *^h^*	**0.11**	**0.083–0.17**				**64 ± 2.4** *^n^*	**0.25**	**0.11–0.38**

*^a^* Taken from ref. [11]. *^b^* Taken from ref. [12]. *^c^* Taken from ref. [13]. *^d^* Decrease in developed tension on isolated guinea-pig left atrium at 5 × 10^−5^ M, expressed as percent changes from the control (*n* = 5–6). The left atria were driven at 1 Hz. *^e^* Calculated from log concentration–response curves (Probit analysis by Litchfield and Wilcoxon [20] with *n* = 6–7). When the maximum effect was <50%, the EC_50_ inotropy and EC_50_ chronotropy values were not calculated. *^f^* Decrease in developed tension on a guinea-pig spontaneously beating isolated right atrium at 10^−5^ M, expressed as percent changes from the control (*n* = 7–8). *^g^* Decrease in atrial rate on a guinea-pig spontaneously beating isolated right atrium at 10^−5^ M, expressed as percent changes from the control (*n* = 7–8). Pretreatment heart rate ranged from 165 to 190 beats/min. The indicated concentrations in *d*, *f*, and *g* produce the maximum effect (M) for most compounds. For compounds that reach the maximum intrinsic activity at concentrations different from those indicated in “*d*”, “*f*”, and “*g*”, the maximum active concentration is indicated with a specific superscripted letter: *^h^* At 10^−5^ M. *^I^* At 10^−6^ M. *^j^* At 5 × 10^−6^ M. *^k^* At 5 × 10^−5^ M. *^l^* At 10^−7^ M. *^m^* At 5 × 10^−5^ M. *^n^* At 5 × 10^−7^ M. **Bold**: unpublished data. SEM: standard error of the mean. Nif (Nifedipine), Lidofl (lidoflazine).

## Supplementary Materials

The following are available online. Table S1: Activity of Tested Compounds on K^+^-depolarized Guinea Pig Vascular Smooth Muscle, NMR spectra of **4e.**
