# Supplementary material for: Cardiovascular Profile of Xanthone-Based 1,4 Dihydropyridines Bearing a Lidoflazine Pharmacophore Fragment
Source: Molecules. 2018 Nov 27;23(12):3088. doi: 10.3390/molecules23123088 (PMC6321116; doi:10.3390/molecules23123088)
Supplement: Supplementary file 1 [file molecules-23-03088-s001.pdf]

Supplementary Materials

Cardiovascular profile of xanthone-based 1,4 dihydropyridines bearing a lidoflazine pharmacophore fragment

Alessandra Bisi*, Matteo Micucci, Silvia Gobbi, Federica Belluti, Roberta Budriesi, Angela Rampa.

Department of Pharmacy and Biotechnology, Alma Mater Studiorum University of Bologna, Via Belmeloro, 6, 40126 Bologna, Italy

Table S1. Activity of Tested Compounds on K^+-depolarized Guinea Pig Vascular Smooth Muscle.

	**Aorta**		
**Cpd**	**Activity[d] (m ± SEM)**	**IC_{50}[e] (μM)**	**95% conf lim ($x10^{-6}$)**
**Nif.**	82 ± 1.3[f]	0.009	0.003−0.02
**Lidofl**	42 ± 1.7[g]		
**1**[a]	**59 ± 4.9**[g]	0.76	0.69–0.85
**2**[a]	7 ± 0.2		
**3a**[b]	51 ± 2.2	**2.18**	**1.88–2.36**
**4a**	**16 ± 0.7**		
**5a**	**20 ± 0.3**		
**3b**[b]	55 ± 1.6	**1.04**	**0.78–1.37**
**4b**	**21 ± 1.3**		
**5b**	**48 ± 2.1**		
**3c**[c]	44 ± 3.4		
**4c**	**6 ± 0.3**		
**5c**	**25 ± 2.0**		
**3d**[c]	31 ± 2.8		
**4d**	**9 ± 0.9**		
**5d**	**36 ± 2.3**		
**3e**[c]	33 ± 2.5		
**4e**	**9 ± 0.3**		
**5e**	**6 ± 0.2**		

[a]Taken from ref 1. [b]Taken from ref 2. [c]Taken from ref 3. [d]Percent inhibition of calcium-induced contraction on K^+-depolarized (80 mM) guinea pig vascular smooth muscle (aortic strips) at $5x10^{-5}$ M. For compounds that reach the maximum intrinsic activity at concentrations different from those indicated in "d" the maximum active concentration is indicated with a specific superscribed letter. [e]Calculated from log concentration-response curves (Probit analysis by Litchfield and Wilcoxon [20]

with n = 6-7). When the maximum effect was <50%, the IC_{50} values were not calculated. [f]At 10^{-6} M. [g]At 10^{-5} M. Bold: unpublished data. Conf lim: confidential limit.

Figure S1. ^{1}H NMR spectra of new compounds**.**

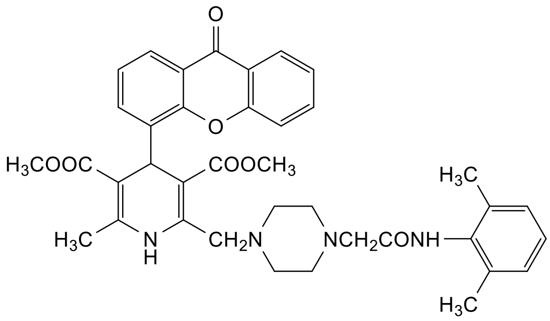

4a

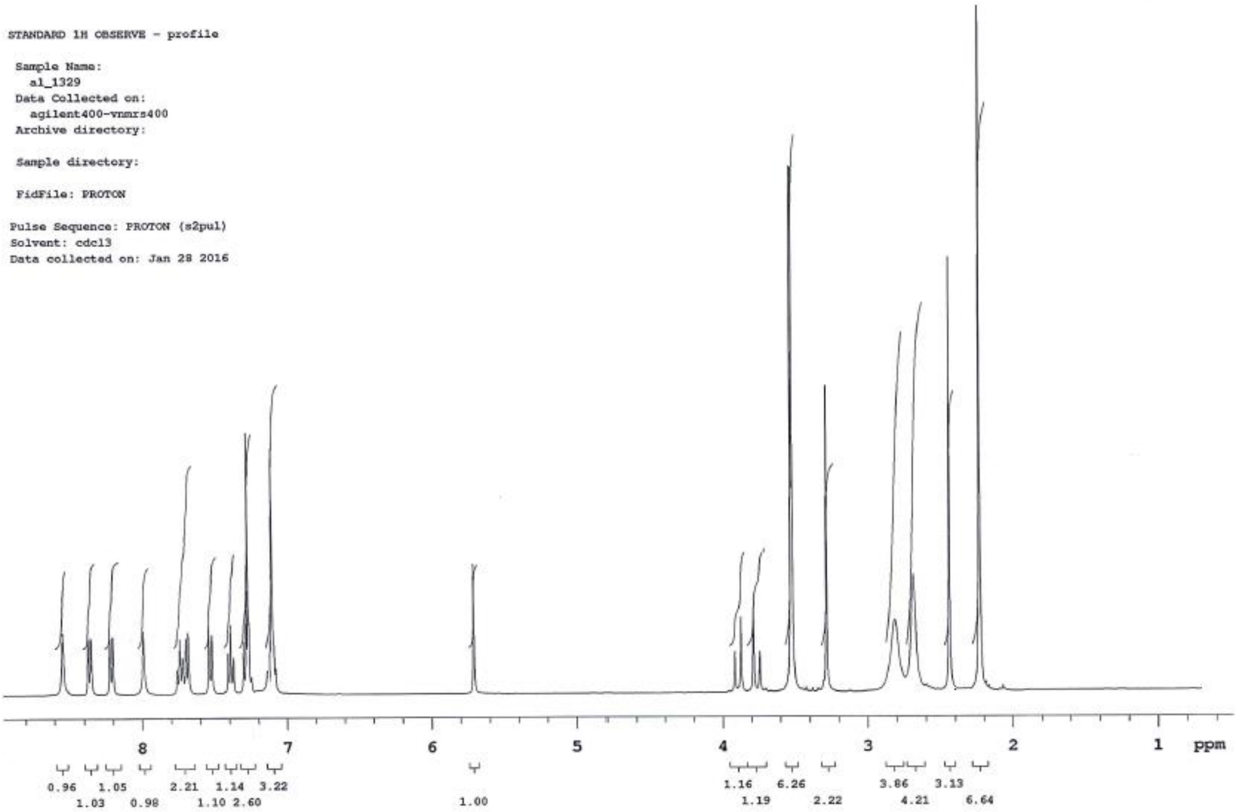

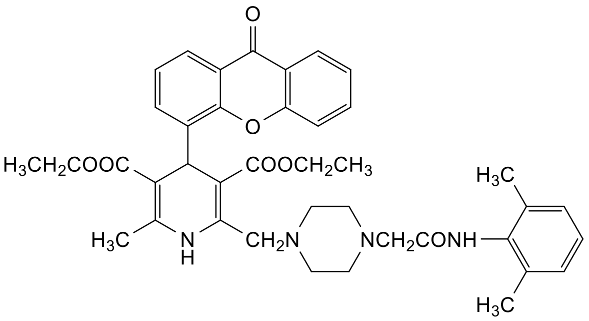
O
H_3CH_2COOC
$COOCH_2CH_3$
H_3C
N
H
CH_2N
NCH_2CONH
H_3C
H_3C

4b

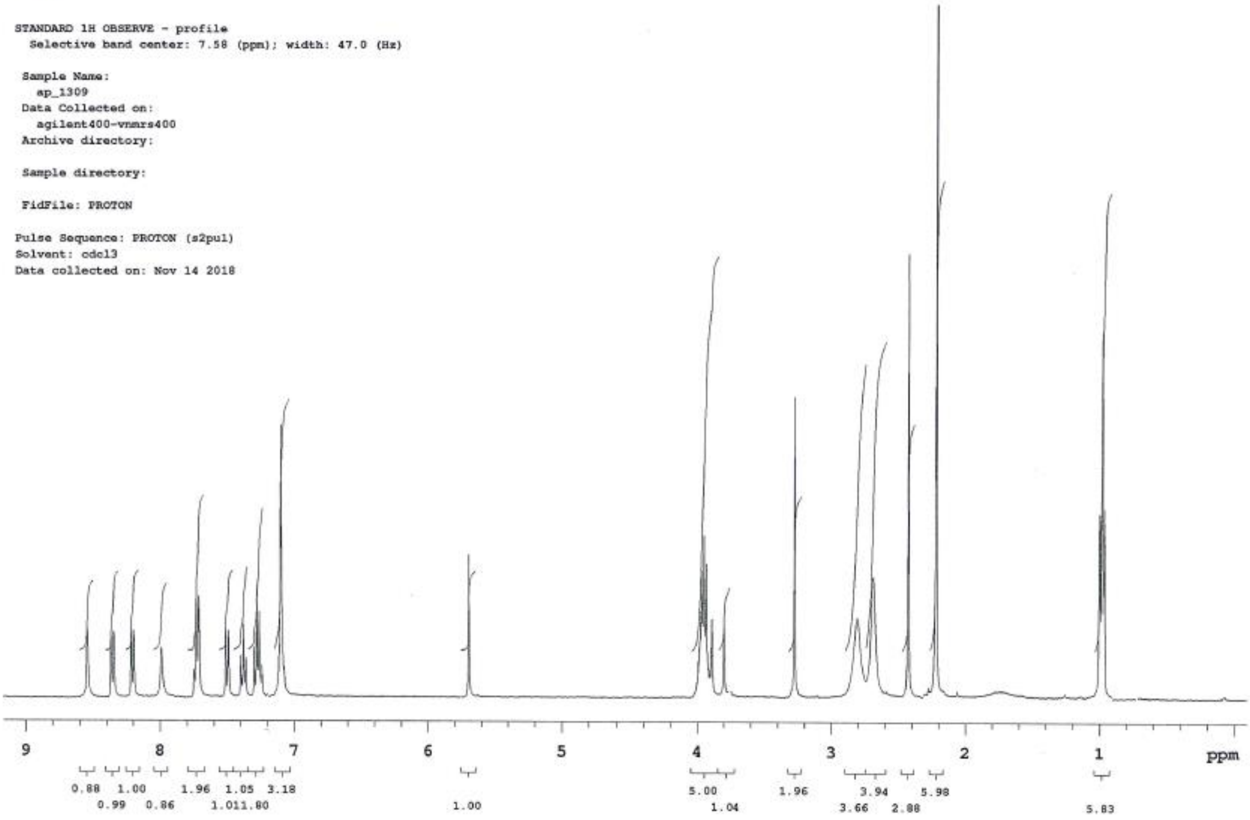
STANDARD 1H OBSERVE - profile
Selective band center: 7.58 (ppm); width: 47.0 (Hz)
Sample Name:
ap_1309
Data Collected on:
agilent400-vnmrs400
Archive directory:
Sample directory:
FidFile: PROTON
Pulse Sequence: PROTON (s2pul)
Solvent: cdcl3
Data collected on: Nov 14 2018
9 8 7 6 5 4 3 2 1 ppm
0.88 1.00 1.96 1.05 3.18
0.99 0.86 1.011.80
1.00
5.00
1.04
1.96
3.94 5.98
3.66 2.88
5.83

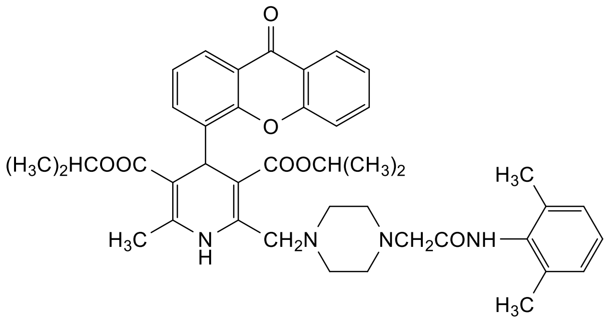
(H3C)2HCOOC
COOCH(CH3)2
H3C
N
H
CH2N
NCH2CONH
H3C
H3C
O

4c

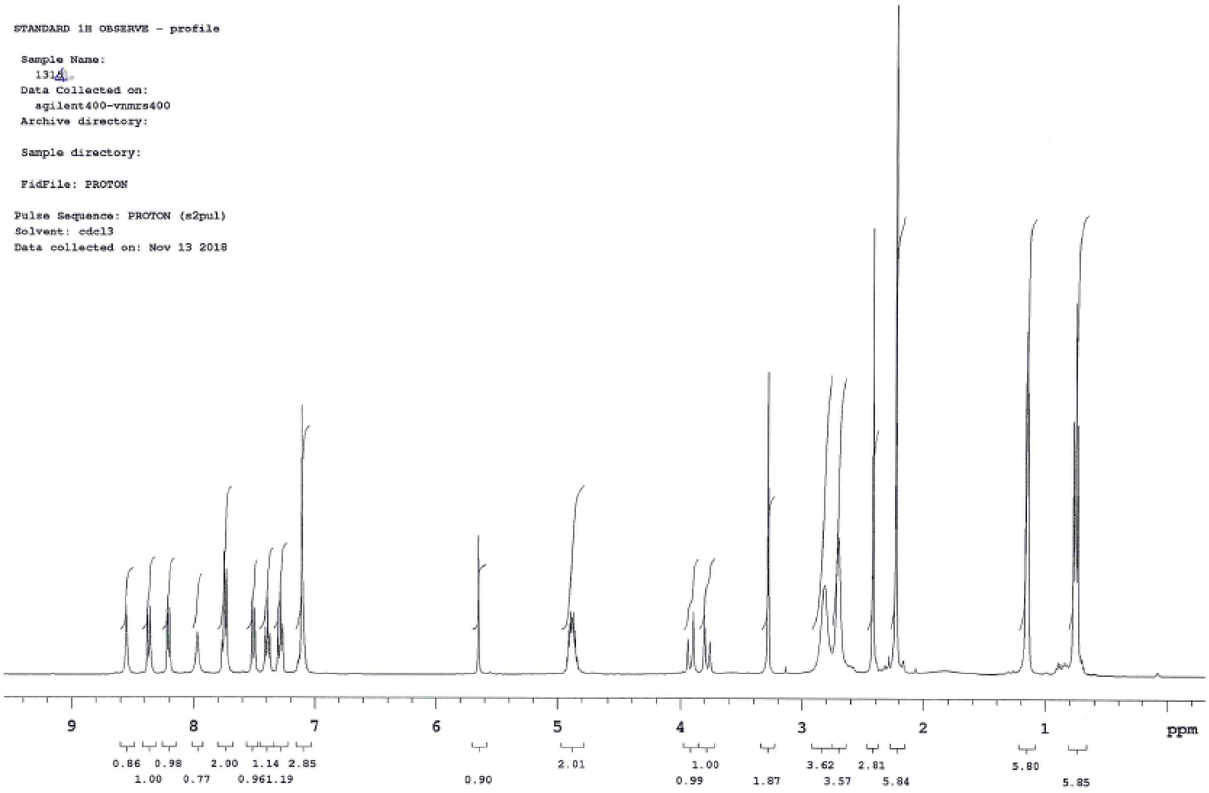
STANDARD 1H OBSERVE - profile
Sample Name:
Data Collected on:
agilent400-vnmrs400
Archive directory:
Sample directory:
FidFile: PROTON
Pulse Sequence: PROTON (s2pul)
Solvent: cdcl3
Data collected on: Nov 13 2018
9
8
7
6
5
4
3
2
1
ppm
0.86 0.98 2.00 1.14 2.85
1.00 0.77 0.961.19
0.90
2.01
1.00
0.99
1.87
3.62 2.81
3.57 5.84
5.80
5.85

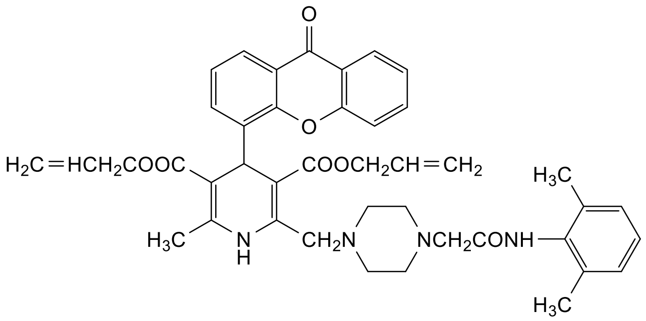
O
O
$H_2C=HCH_2COOC$
$COOCH_2CH=CH_2$
H_3C
H_3C
N
H
CH_2N
NCH_2CONH
H_3C

4d

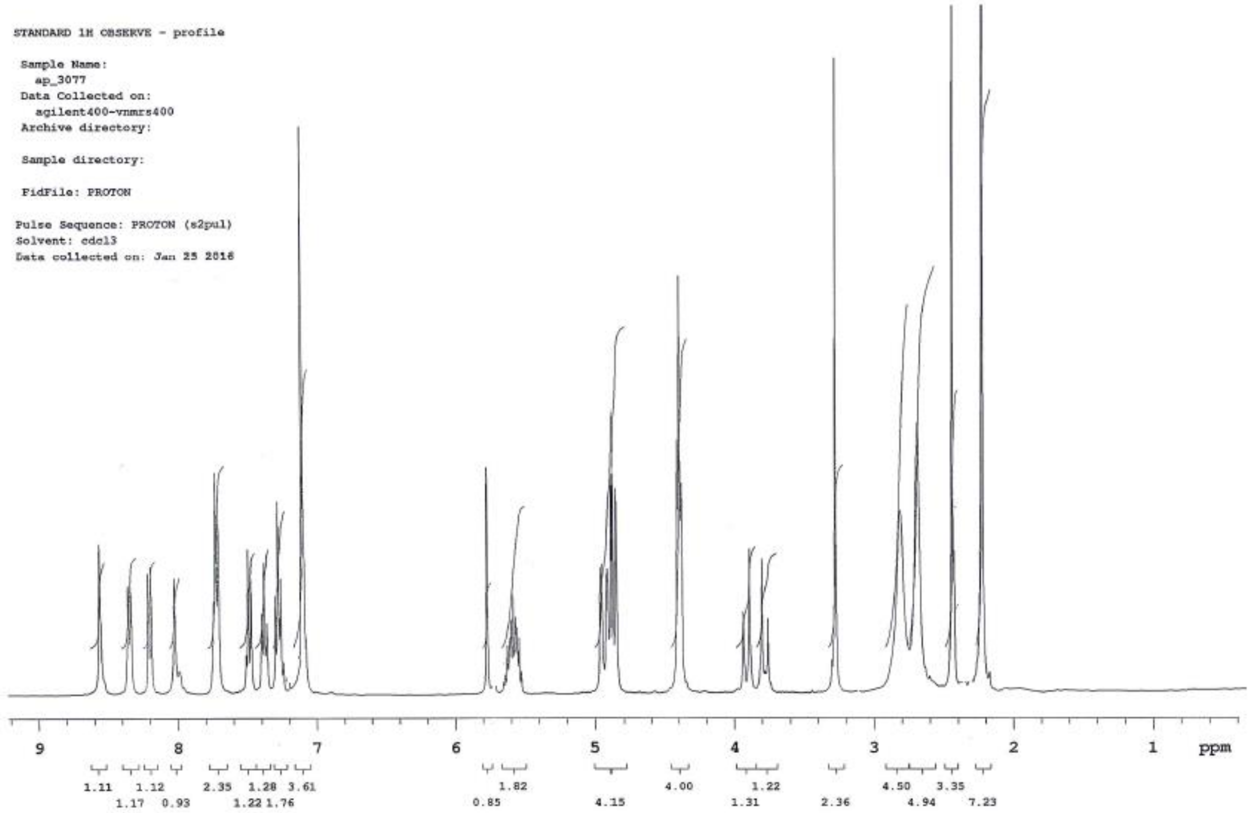
STANDARD 1H OBSERVE - profile
Sample Name:
ap_3077
Data Collected on:
agilent400-vnmrs400
Archive directory:
Sample directory:
FidFile: PROTON
Pulse Sequence: PROTON (s2pul)
Solvent: cdcl3
Data collected on: Jan 25 2016
9
8
7
6
5
4
3
2
1
ppm
1.11
1.17
1.12
0.93
2.35
1.22
1.28
1.76
3.61
0.85
1.82
4.15
4.00
1.31
1.22
2.36
4.50
4.94
3.35
7.23

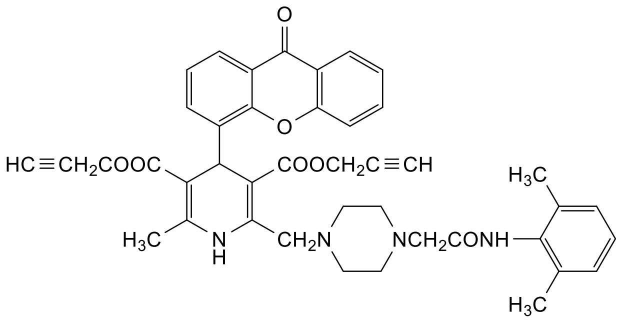

4e

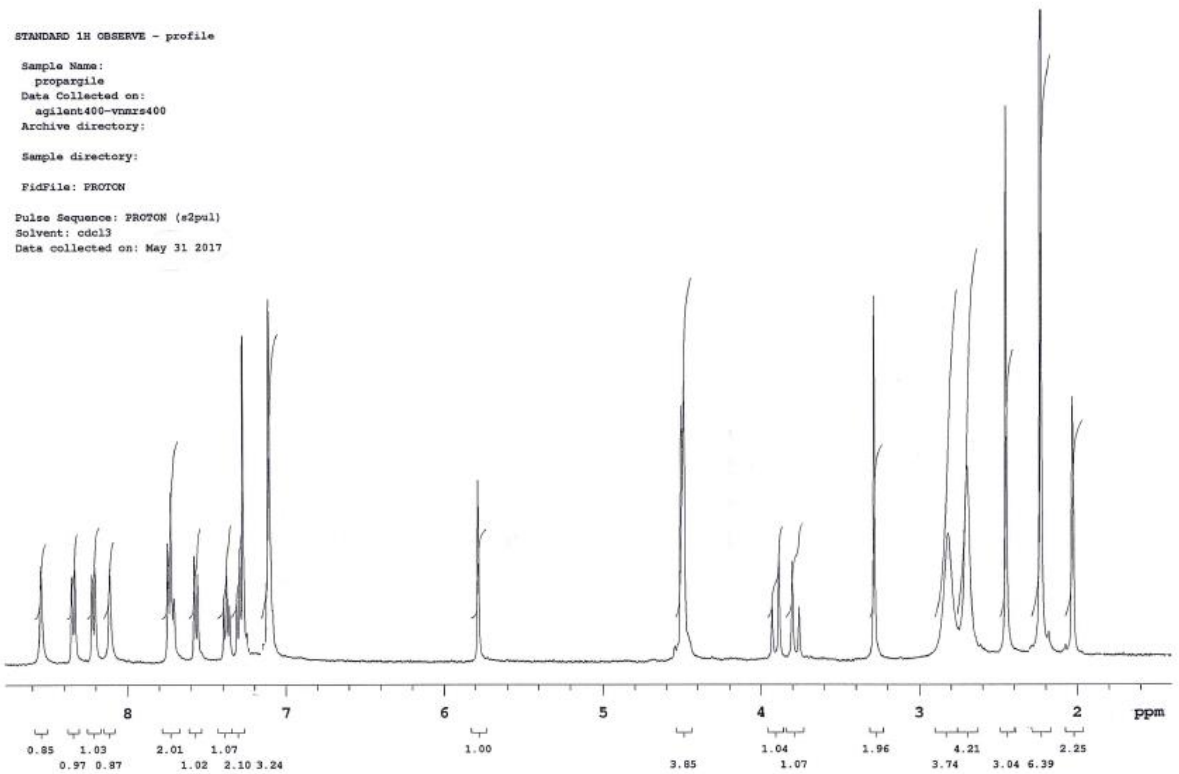

STANDARD 1H OBSERVE - profile
Sample Name:
propargile
Data Collected on:
agilent400-vnmrs400
Archive directory:
Sample directory:
FidFile: PROTON
Pulse Sequence: PROTON (s2pul)
Solvent: cdcl3
Data collected on: May 31 2017
8 7 6 5 4 3 2 ppm
0.85 0.97 1.03 0.87 2.01 1.02 1.07 2.10 3.24 1.00 3.85 1.04 1.07 1.96 3.74 4.21 3.04 6.39 2.25

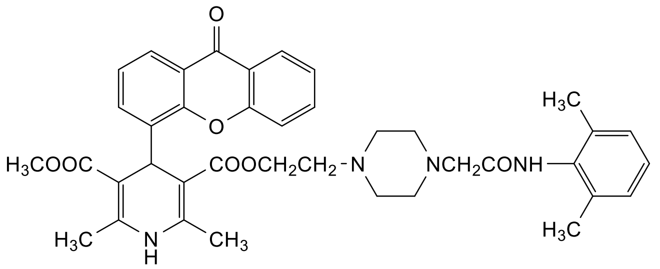
O
O
H3COOC
COOCH2CH2
N
NCH2CONH
H3C
H3C
N
H
CH3
H3C

5a

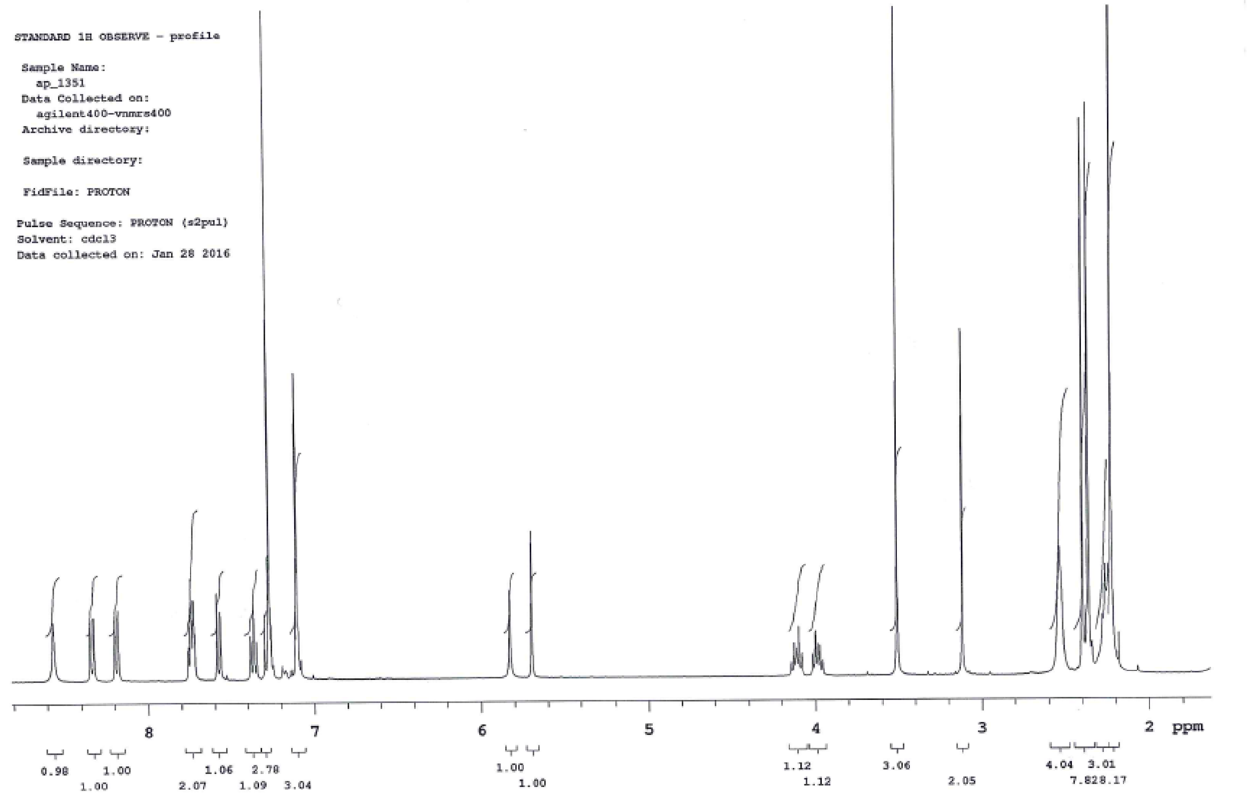
STANDARD 1H OBSERVE - profile
Sample Name:
ap_1351
Data Collected on:
agilent400-vnmrs400
Archive directory:
Sample directory:
FidFile: PROTON
Pulse Sequence: PROTON (s2pul)
Solvent: cdcl3
Data collected on: Jan 28 2016
8
7
6
5
4
3
2 ppm
0.98
1.00
1.00
2.07
1.06
1.09
2.78
3.04
1.00
1.00
1.12
1.12
3.06
2.05
4.04
3.01
7.828.17

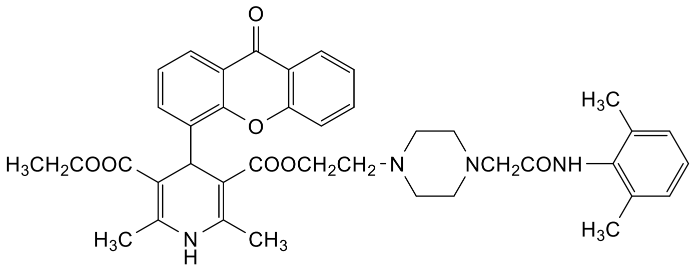
O
O
H3CH2COOC
COOCH2CH2
N
NCH2CONH
H3C
H3C
CH3
N
H
H3C

5b

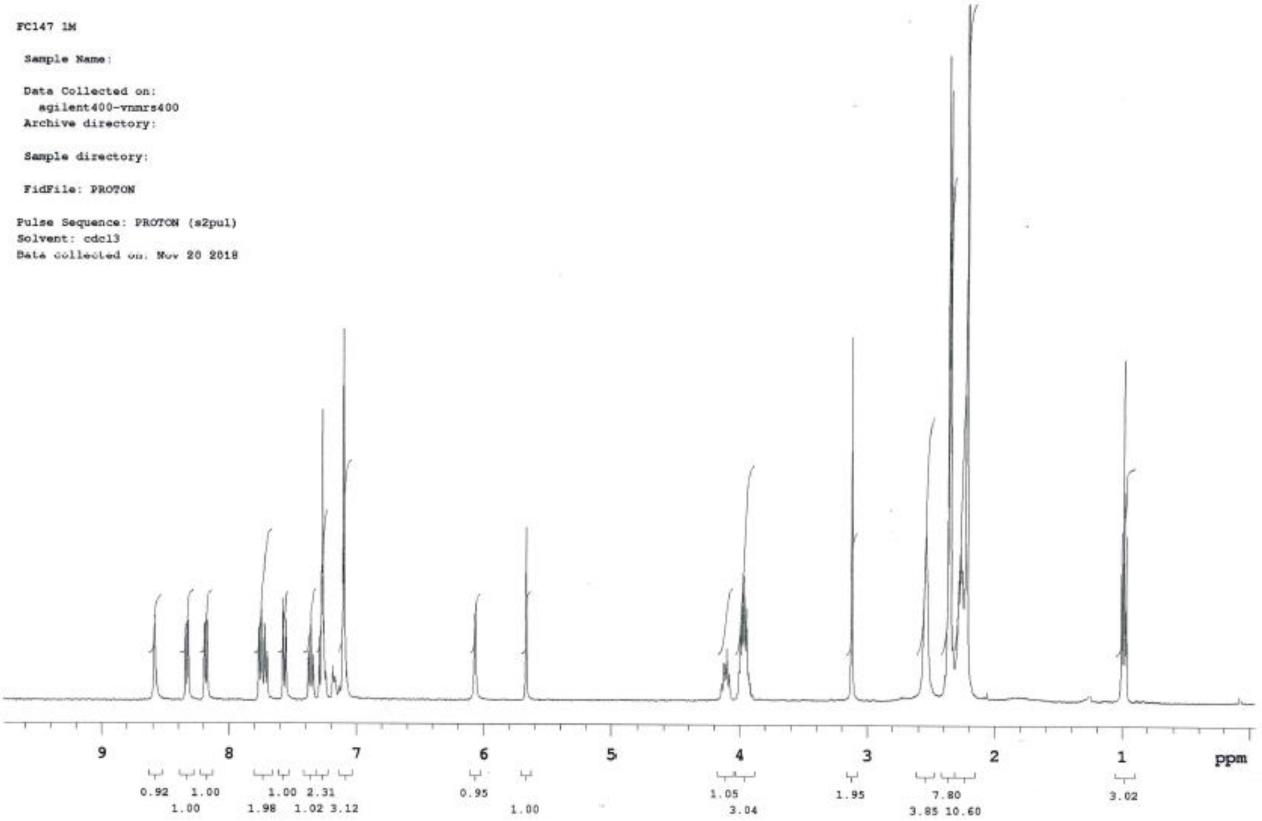
FC147 1H
Sample Name:
Data Collected on:
agilent400-vnmrs400
Archive directory:
Sample directory:
FidFile: PROTON
Pulse Sequence: PROTON (s2pul)
Solvent: cdcl3
Data collected on: Nov 20 2018
9
8
7
6
5
4
3
2
1
ppm
0.92
1.00
1.00
1.00
1.98
1.00
2.31
1.02
3.12
0.95
1.00
1.05
3.04
1.95
7.80
3.85
10.60
3.02

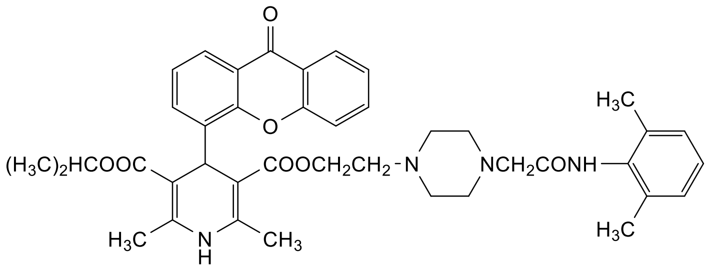
O
(H3C)2HCOOC
COOCH2CH2
N
NCH2CONH
H3C
CH3
H

5c

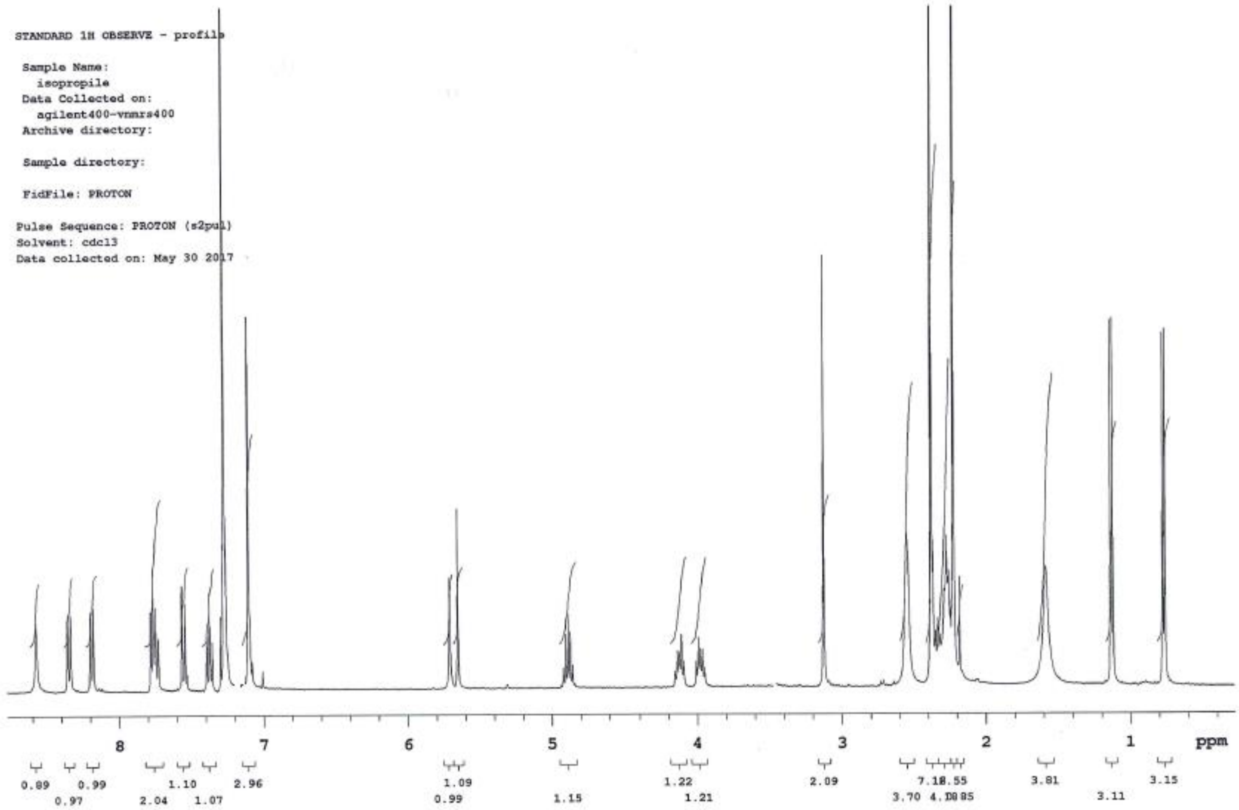
STANDARD 1H OBSERVE - profile
Sample Name:
isopropile
Data Collected on:
agilent400-vnmrs400
Archive directory:
Sample directory:
FidFile: PROTON
Pulse Sequence: PROTON (s2pul)
Solvent: cdcl3
Data collected on: May 30 2017
8
7
6
5
4
3
2
1
ppm
0.89
0.97
0.99
2.04
1.10
1.07
2.96
1.09
0.99
1.15
1.22
1.21
2.09
3.70
3.81
3.11
3.15

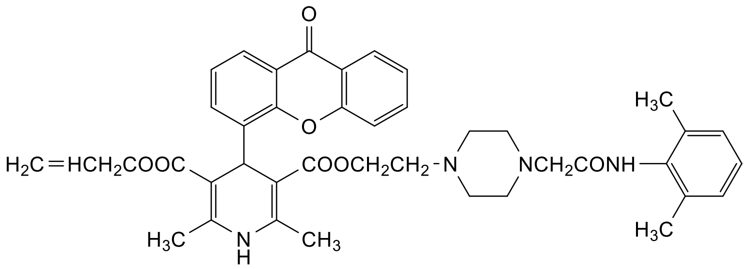
O
H3C
H2C=HCH2COOC
COOCH2CH2-N
NCH2CONH
H3C
N
H
CH3
H3C

5d

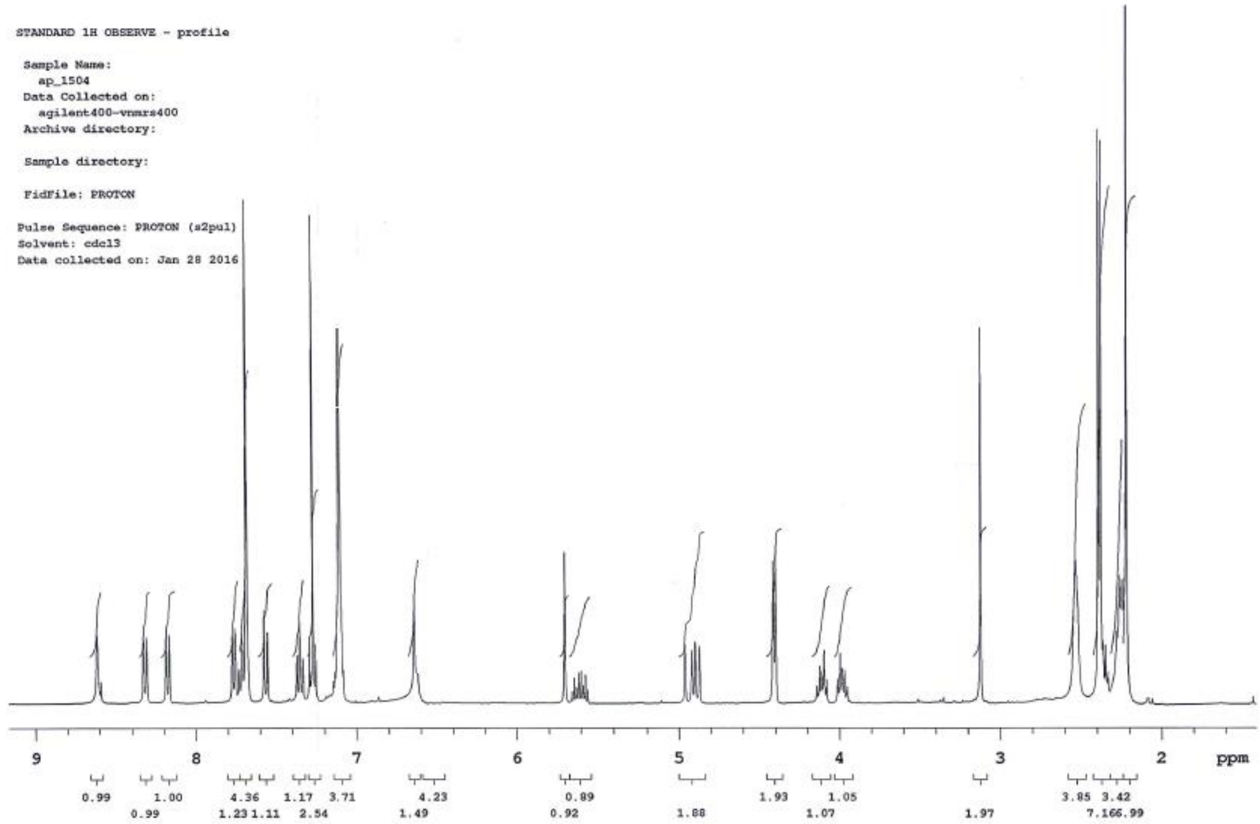
STANDARD 1H OBSERVE - profile
Sample Name:
ap_1504
Data Collected on:
agilent400-vnmrs400
Archive directory:
Sample directory:
FidFile: PROTON
Pulse Sequence: PROTON (s2pul)
Solvent: cdcl3
Data collected on: Jan 28 2016
9
8
7
6
5
4
3
2
ppm
0.99
0.99
1.00
4.36
1.23
1.11
1.17
2.54
3.71
4.23
1.49
0.89
0.92
1.88
1.93
1.07
1.05
1.97
3.85
3.42
7.16
6.99

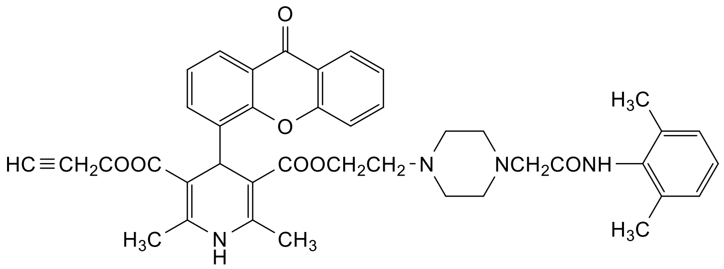

5e

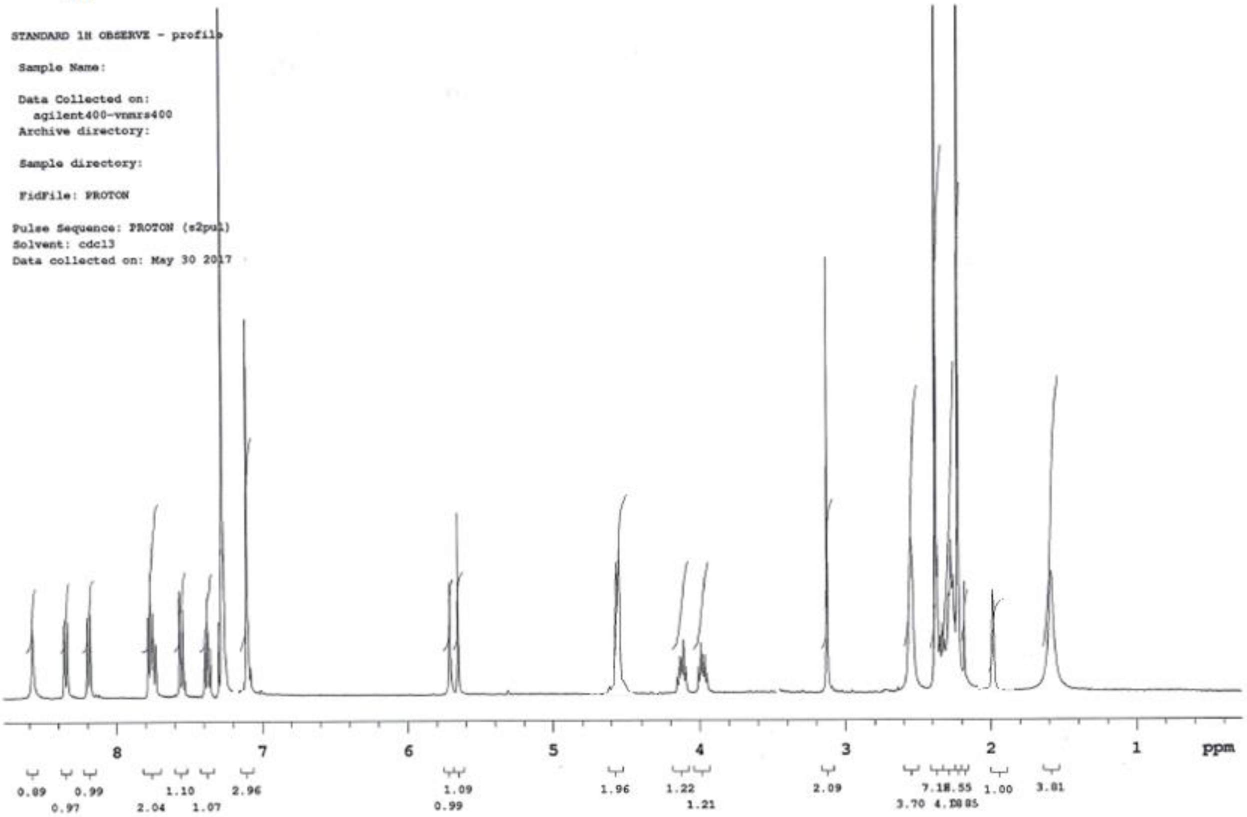

Figure S2. ^{13}C NMR spectra of new compounds.

4a

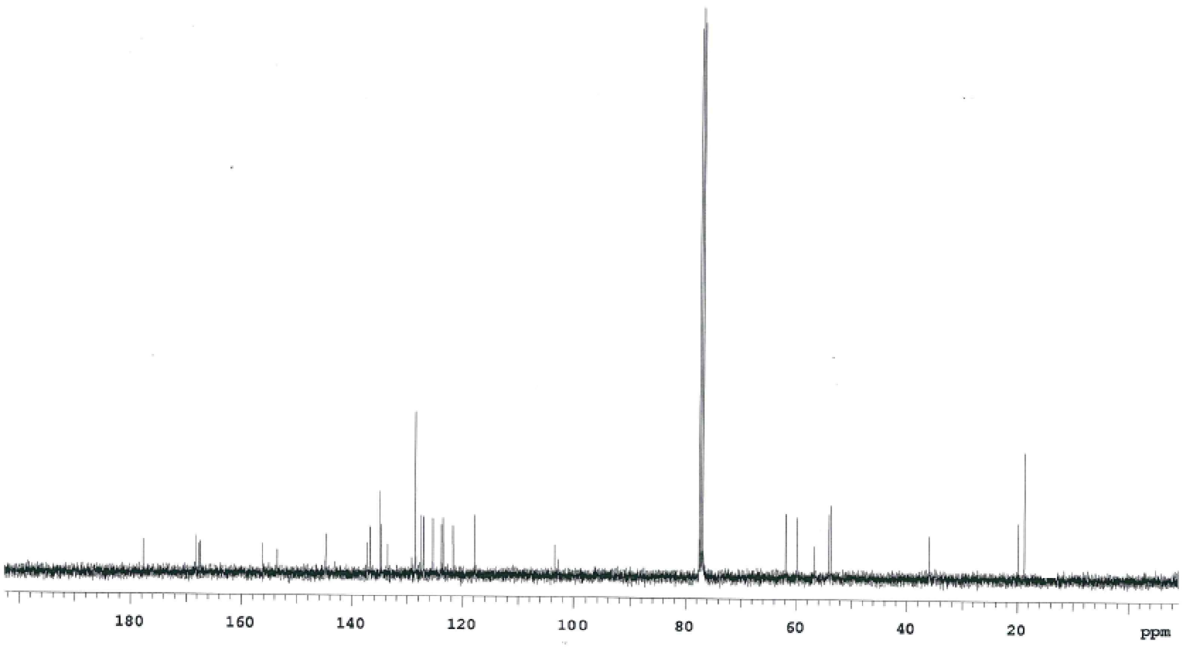

4b

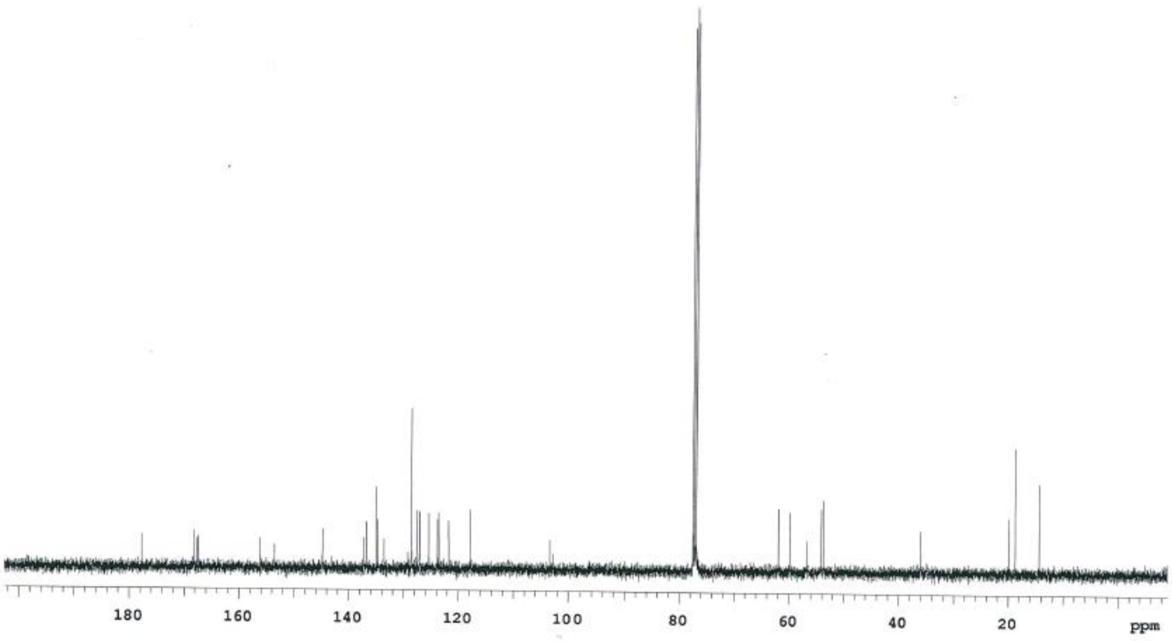

4c

STANDARD 1H OBSERVE - profile

Sample Name:
131[illegible]
Data Collected on:
agilent400-vnmrs400
Archive directory:

Sample directory:

FidFile: CARBON

Pulse Sequence: CARBON (s2pul)
Solvent: cdcl3
Data collected on: Nov 13 2018

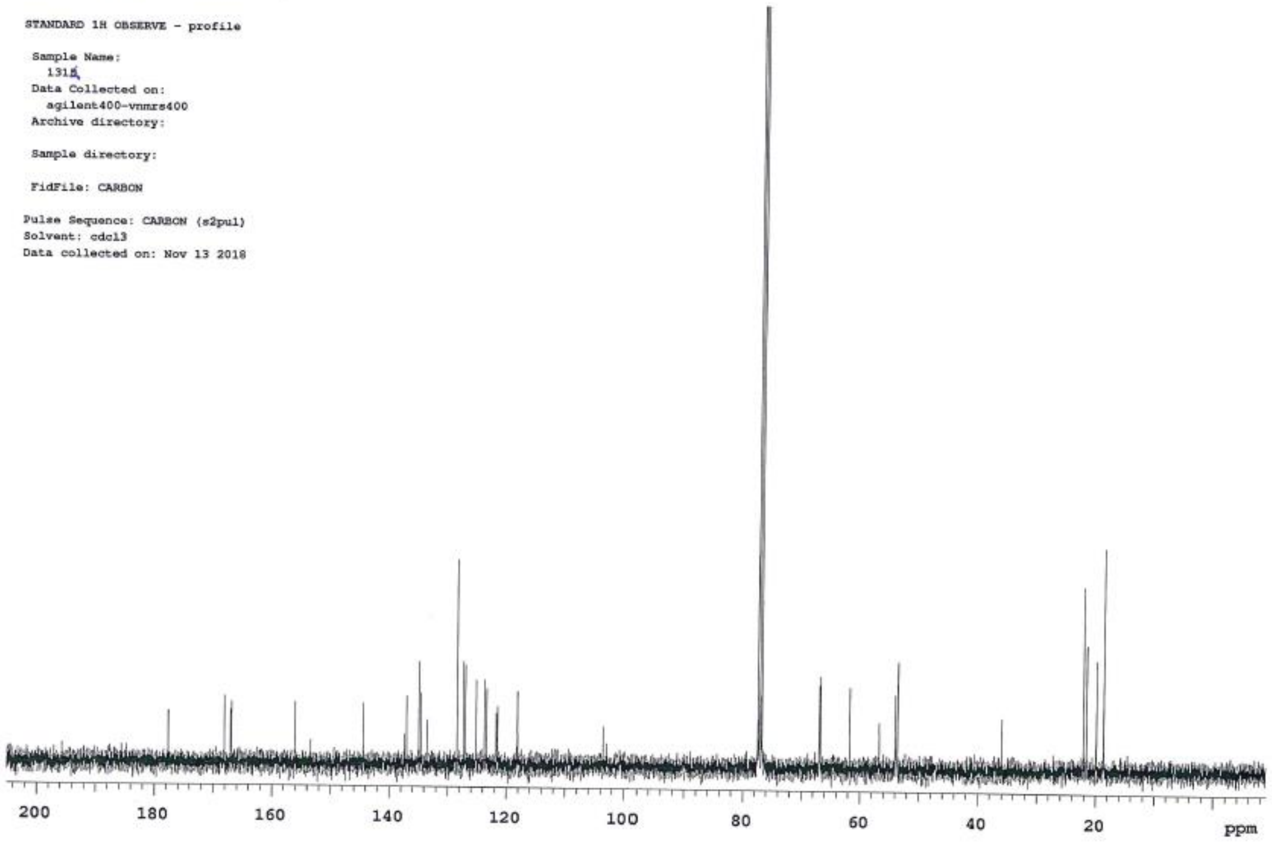

4d

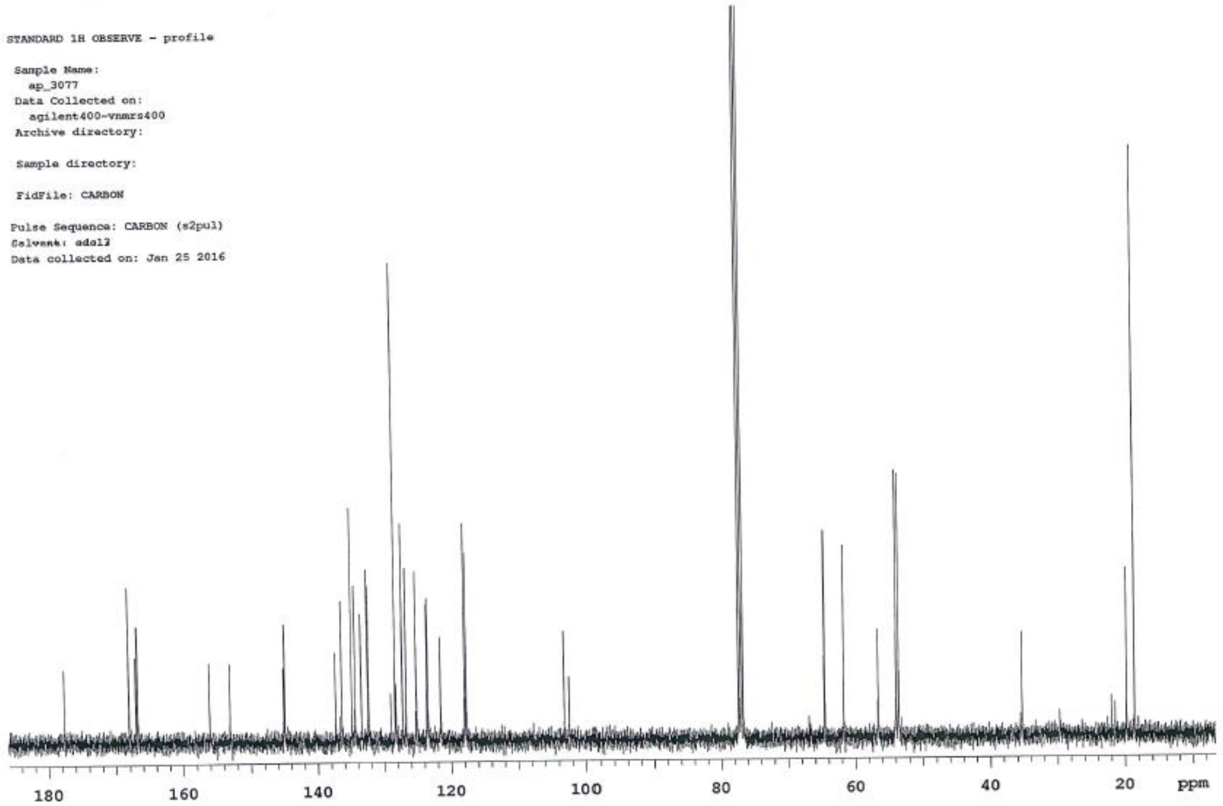
STANDARD 1H OBSERVE - profile
Sample Name:
ap_3077
Data Collected on:
agilent400-vnmrs400
Archive directory:
Sample directory:
FidFile: CARBON
Pulse Sequence: CARBON (s2pul)
Solvent: cdcl3
Data collected on: Jan 25 2016
180
160
140
120
100
80
60
40
20
ppm

4e

STANDARD 1H OBSERVE - profile

Sample Name:
propargile
Data Collected on:
agilent400-vnmrs400
Archive directory:

Sample directory:

FidFile: CARBON

Pulse Sequence: CARBON (s2pul)
Solvent: cdcl3
Data collected on: May 31 2017

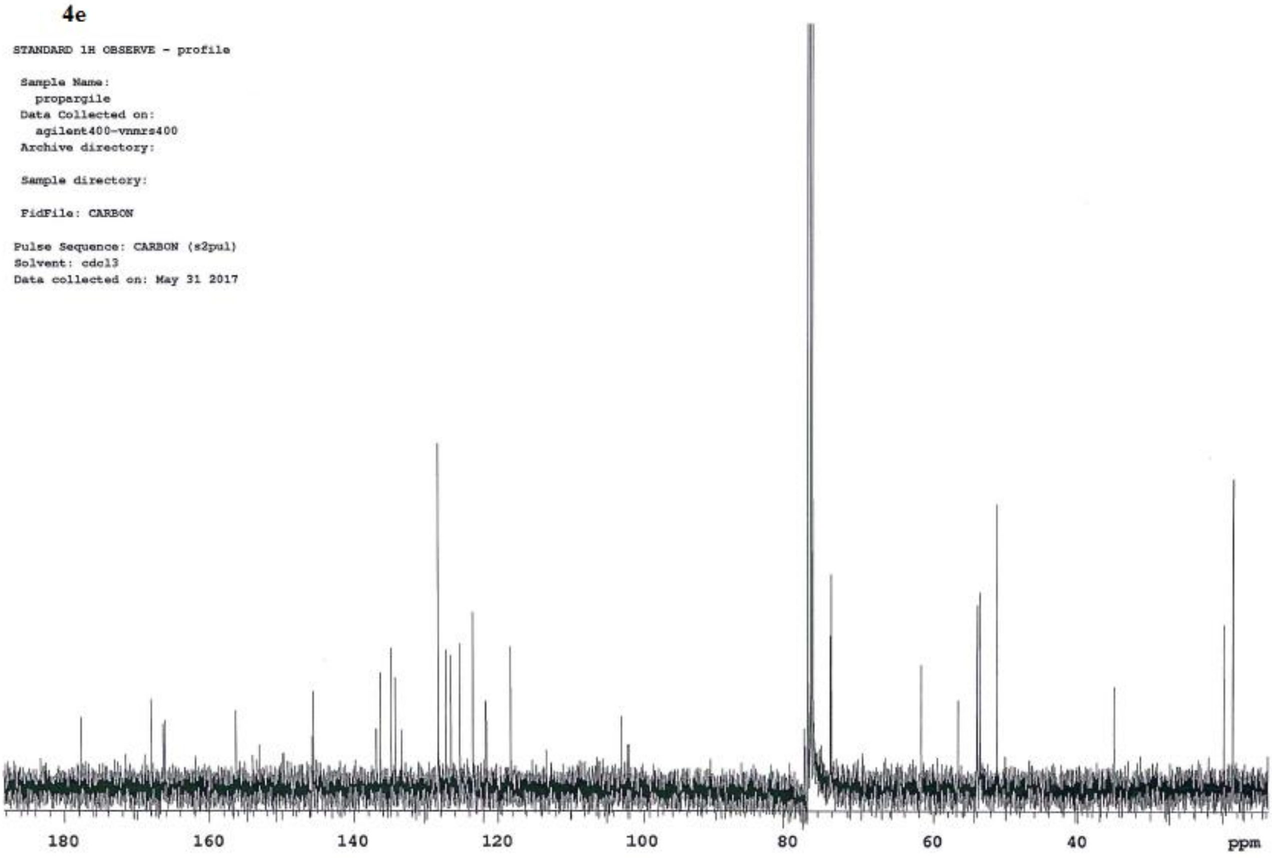

5a

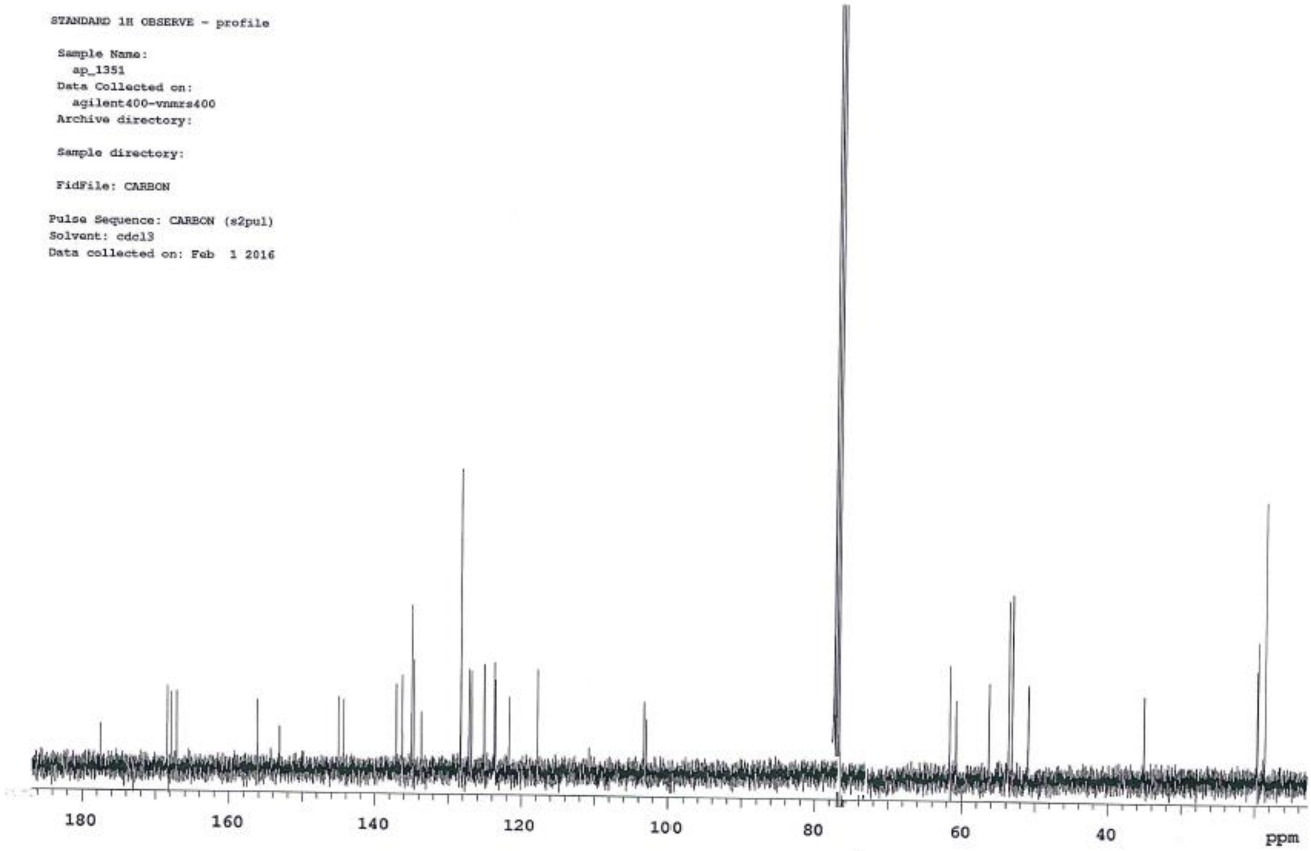
STANDARD 1H OBSERVE - profile
Sample Name:
ap_1351
Data Collected on:
agilent400-vnmrs400
Archive directory:
Sample directory:
FidFile: CARBON
Pulse Sequence: CARBON (s2pul)
Solvent: cdcl3
Data collected on: Feb 1 2016
180
160
140
120
100
80
60
40
ppm

5b

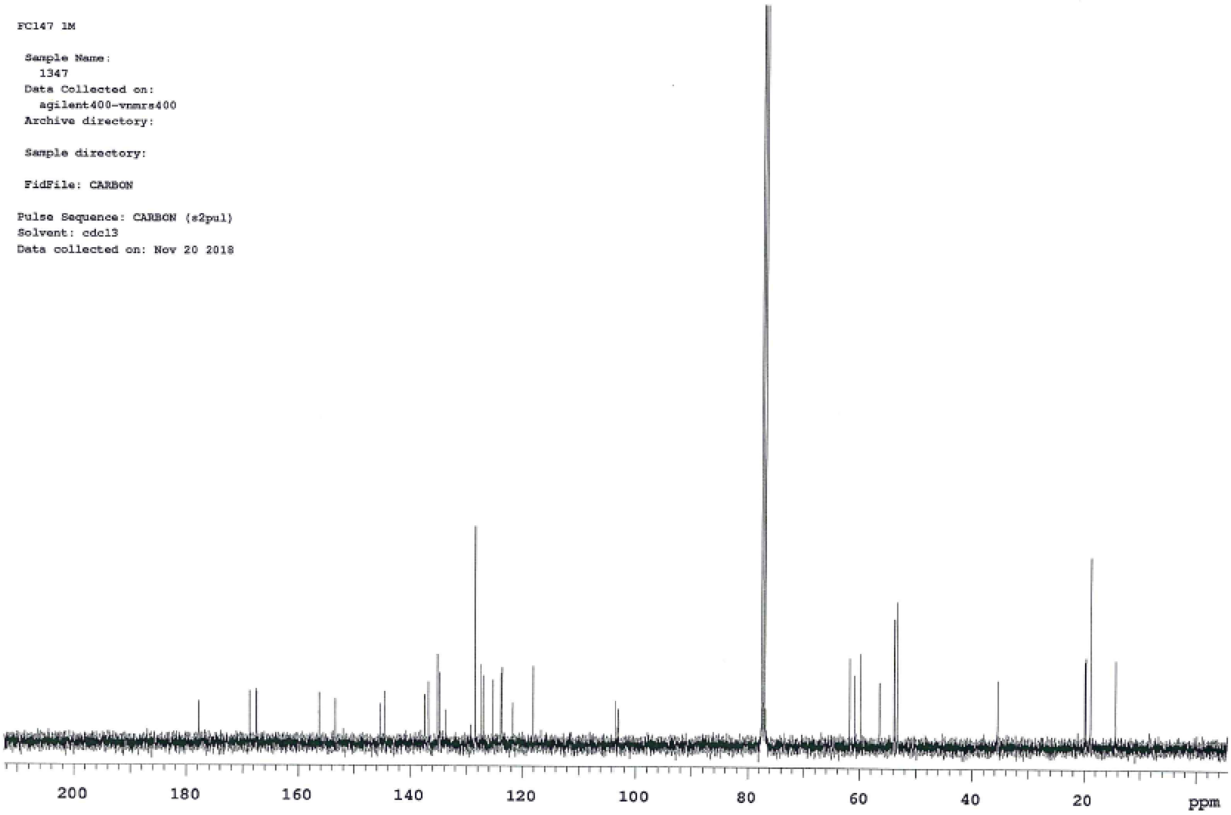
FC147 1M
Sample Name:
1347
Data Collected on:
agilent400-vnmrs400
Archive directory:
Sample directory:
FidFile: CARBON
Pulse Sequence: CARBON (s2pul)
Solvent: cdcl3
Data collected on: Nov 20 2018
200
180
160
140
120
100
80
60
40
20
ppm

5c

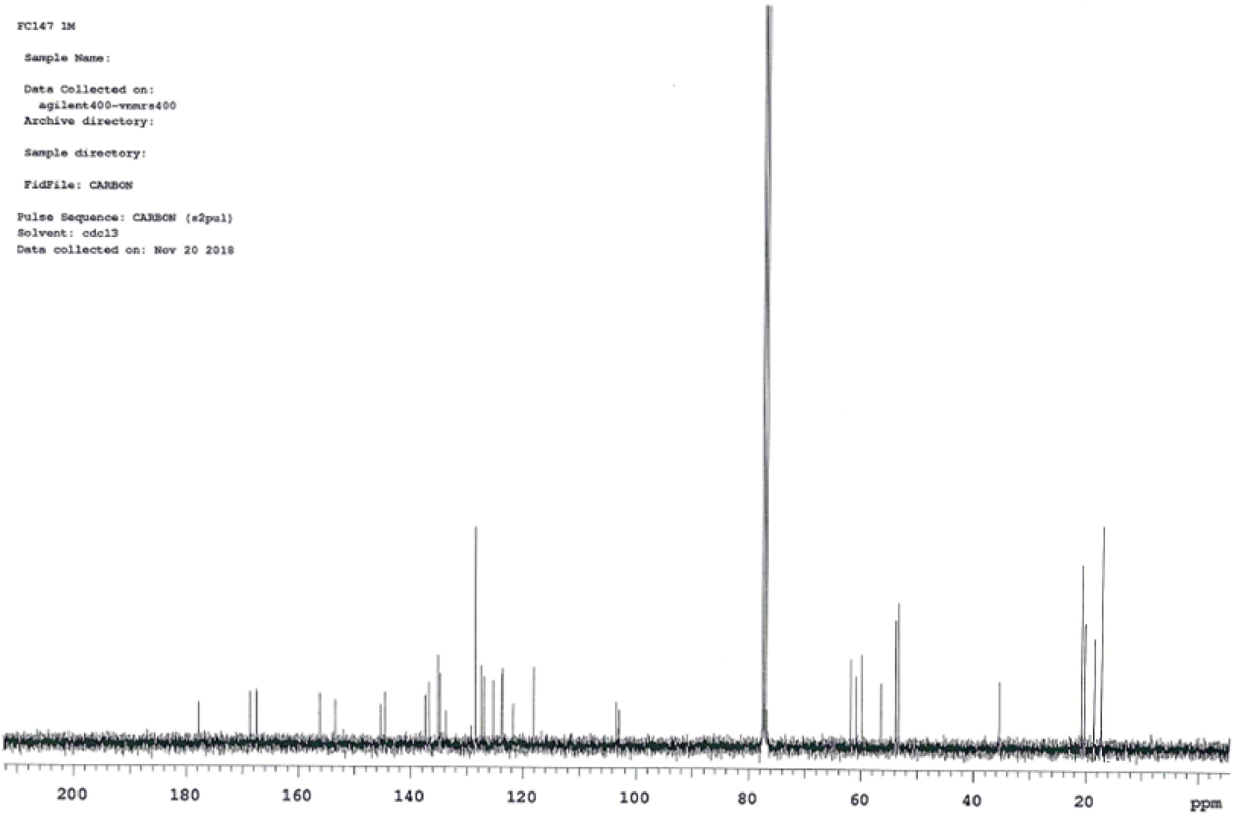
FC147 1M
Sample Name:
Data Collected on:
agilent400-vnmrs400
Archive directory:
Sample directory:
FidFile: CARBON
Pulse Sequence: CARBON (s2pul)
Solvent: cdcl3
Data collected on: Nov 20 2018
200 180 160 140 120 100 80 60 40 20
ppm

5d

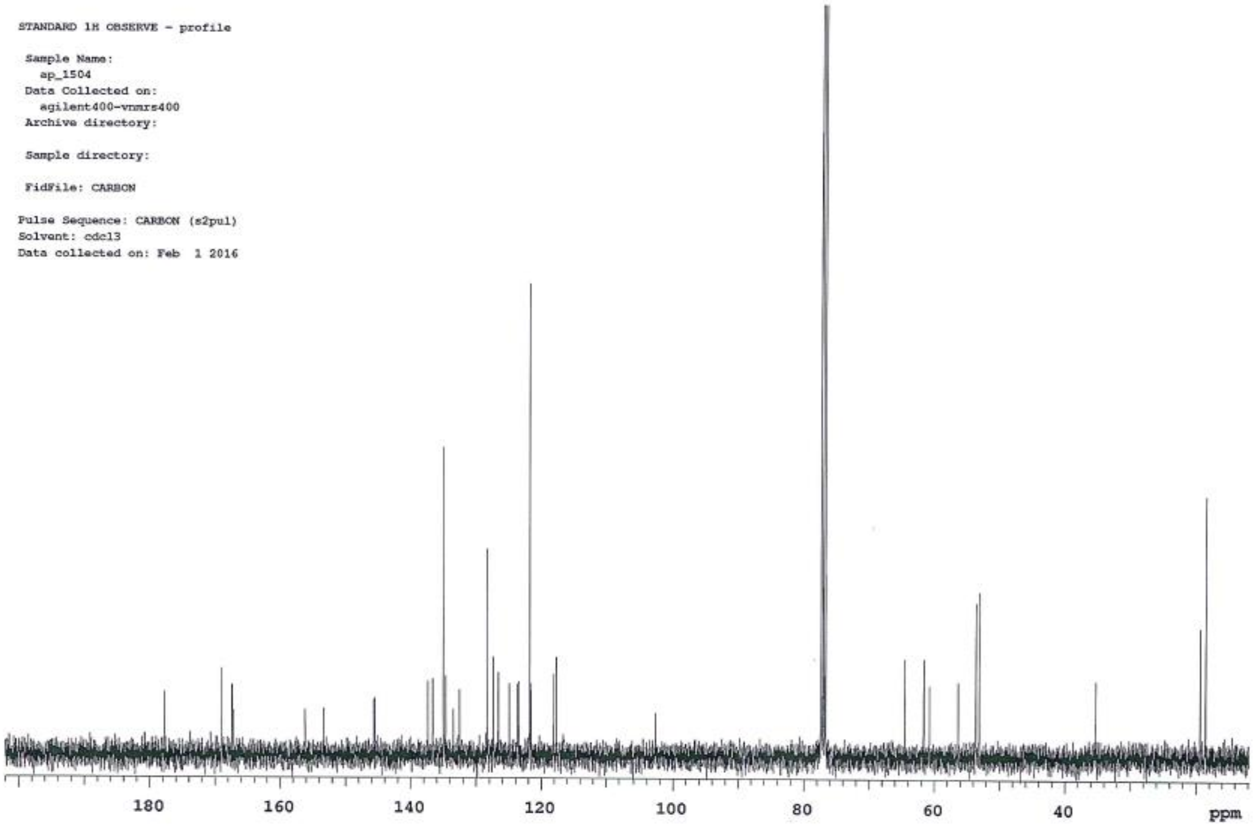
STANDARD 1H OBSERVE - profile
Sample Name:
ap_1504
Data Collected on:
agilent400-vnmrs400
Archive directory:
Sample directory:
FidFile: CARBON
Pulse Sequence: CARBON (s2pul)
Solvent: cdcl3
Data collected on: Feb 1 2016
180
160
140
120
100
80
60
40
ppm

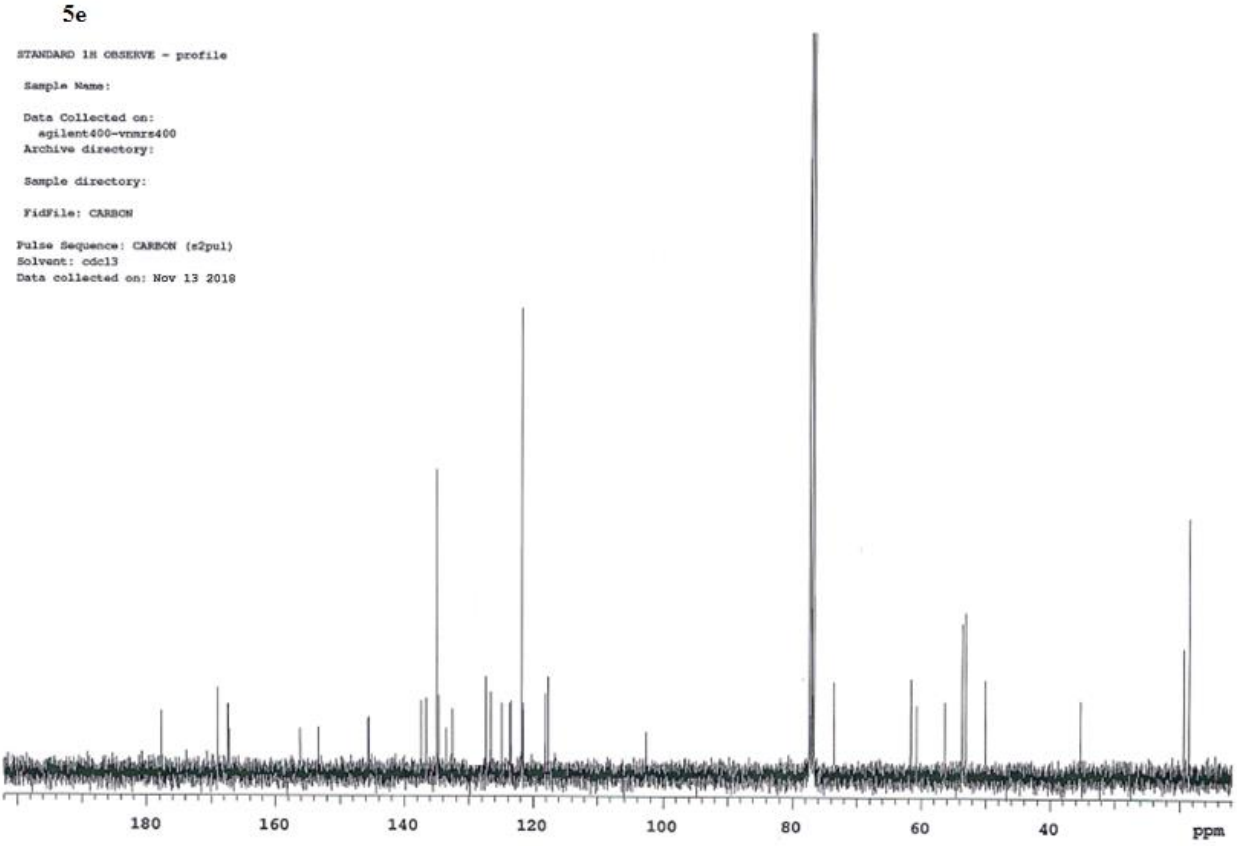

References

1. Chiarini A, Rampa A, Budriesi R, Bisi A, Fabbri G, Valenti P. 1,4-Dihydropyridines bearing a pharmacophoric fragment of lidoflazine. *Bioorg Med Chem*. 1996; 10:1629-1635.

2. Valenti P, Chiarini A, Gasperi F, Budriesi R. Xanthone 1,4-dihydropyridine derivatives with a potent selective bradycardic effect. *Arzneimittelforschung*. 1990; 40:122-125.

3. Chiarini A, Rampa A, Bisi A, Budriesi R, Valenti P. Negative inotropic and chronotropic activity of calcium channel ligands possessing a xanthone 1,4-dihydropyridine backbone. *Arzneimittelforschung*. 1992; 42:797-801.
